# Comprehensive clinical assays for molecular diagnostics of gliomas: the current state and future prospects

**DOI:** 10.3389/fmolb.2023.1216102

**Published:** 2023-10-16

**Authors:** Alina Penkova, Olga Kuziakova, Valeriia Gulaia, Vladlena Tiasto, Nikolay V. Goncharov, Daria Lanskikh, Valeriia Zhmenia, Ivan Baklanov, Vladislav Farniev, Vadim Kumeiko

**Affiliations:** ^1^ Institute of Life Sciences and Biomedicine, Far Eastern Federal University, Vladivostok, Russia; ^2^ A. V. Zhirmunsky National Scientific Center of Marine Biology, FEB RAS, Vladivostok, Russia

**Keywords:** cancer molecular diagnostics, NGS panel, PCR, molecular markers of glioma, medical market

## Abstract

Glioma is one of the most intractable types of cancer, due to delayed diagnosis at advanced stages. The clinical symptoms of glioma are unclear and due to a variety of glioma subtypes, available low-invasive testing is not effective enough to be introduced into routine medical laboratory practice. Therefore, recent advances in the clinical diagnosis of glioma have focused on liquid biopsy approaches that utilize a wide range of techniques such as next-generation sequencing (NGS), droplet-digital polymerase chain reaction (ddPCR), and quantitative PCR (qPCR). Among all techniques, NGS is the most advantageous diagnostic method. Despite the rapid cheapening of NGS experiments, the cost of such diagnostics remains high. Moreover, high-throughput diagnostics are not appropriate for molecular profiling of gliomas since patients with gliomas exhibit only a few diagnostic markers. In this review, we highlighted all available assays for glioma diagnosing for main pathogenic glioma DNA sequence alterations. In the present study, we reviewed the possibility of integrating routine molecular methods into the diagnosis of gliomas. We state that the development of an affordable assay covering all glioma genetic aberrations could enable early detection and improve patient outcomes. Moreover, the development of such molecular diagnostic kits could potentially be a good alternative to expensive NGS-based approaches.

## 1 Introduction

Glioma is one of the most common forms of malignant tumors of the central nervous system that grow from glial cells in the brain and spinal cord. Glioma can diffusely infiltrate and affect surrounding brain tissue, which determines the many dramatic consequences for patients who have the disease. The manifestation of glioma is most characteristic for people aged over 50 years, is rare for those aged up to 30 years, and is distinctive for the male part of the population (Yang et al., 2022). The incidence of brain tumors per 100,000 people differs from region to region—the incidence is higher in Europe (5.5), North America (5.3), Australia/New Zealand (5.3), Western Asia (5.2), and Northern Africa (5.0). The lowest incidence is in South-Central Asia (1.8), sub-Saharan Africa (0.8), and Oceania excluding Australia and New Zealand (0.5). However, these variations can be explained as true differences in the number of cases or an influence of the data collection method (Ostrom et al., 2015). Every year 80.000 primary cases of brain tumors are diagnosed in the United States with approximately 25% accounting for gliomas. The total number of glioblastoma cases annually is 12.000 (Mesfin and Al-Dhahir, 2022).

In 2016, the World Health Organization classified central nervous system tumors (CNS WHO 4) using molecular markers. Moreover, the updated WHO classification 2021 (WHO CNS 5) gives even higher priority to molecular diagnostics. As a result, new types of tumors were included in the classification. According to the latest WHO CNS5 2021, the prognosis and treatment of gliomas can vary significantly, depending on the glioma type (Louis et al., 2021). That is why it is necessary to develop universal diagnostic test systems to differentiate the diagnosis and select the appropriate treatment for gliomas. Nowadays, standard diagnostic methods that are based on histological examination remain not accurate due to the absence of a uniform evaluation method. Moreover, accurate histological classification can be hampered by insufficient or unrepresentative tissue samples (Coons et al., 1997; Nikiforova and Hamilton, 2011). To make a proper glioma molecular profiling, a modern laboratory infrastructure requires the establishment of several separated facilities, as its current classification requires fluorescence *in situ* hybridization (FISH) to detect large chromosomal aberrations, overexpression, loss-of-function analysis using immunohistochemistry (IHС), gene fusions examined by quantitative PCR (qPCR), single nucleotide substitutions/polymorphisms (SNPs) addressed by Sanger sequencing, and finally gene/promoter methylation studied by pyrosequencing. Only proper molecular characterization of glioma samples would result in better clinical management and realistic prognosis for the disease, as well as collecting representative data for researchers seeking the possible key to care.

Furthermore, one should not forget that there is a variety of glioma biomarkers that could be used for non-invasive diagnostics other than DNA sequence. The development of methods of diagnosis based on proteomics, lipidomics, and metabolomics was discussed recently (Gaca-Tabaszewska et al., 2022; Pienkowski et al., 2022). It was found that there is a metabolic difference in glioma with mutant or wild-type IDH1, with or without 1p/19q-codeletion (Goryńska et al., 2022). However, the use of such biomarkers has its limitations. They cannot be used to distinguish between the molecular subtypes of glioma or to monitor the genetic evolution of the tumor or its heterogeneity.

Since the clinical market still lacks a universal screening/diagnostic glioma assay based on one method and incorporating all molecular markers, we have reviewed all possible methods that could be applied to create such a diagnostic system. In this review, we list the commercially available NGS/PCR/FISH kits and describe their utility and suitability for glioma molecular profiling. Researchers can refer to this paper for developing screening or diagnostic glioma panels and clinicians can use it to choose a better available system.

In this study we are trying to summarize and review the existing methods for the diagnosis of glioma with the terms “*IDH1* molecular diagnostic,” “glioma molecular diagnostic,” “*1p/19q* molecular diagnostic,” “(gene name)” PCR, “glioma NGS panel,” and “(gene name)” NGS panel. Furthermore, we have highlighted new test systems that are used in clinical labs in different parts of the world and some promising methods. We analyzed the availability of next-generation sequencing (NGS) platforms globally by discovering national research centers in different countries.

In summary, despite NGS being the most sensitive and specific method to detect mutations in glioma and monitor its recurrence, there are many obstacles to making this technology universal in clinical diagnosis. Therefore, the requirement for a molecular diagnostic system could be solved by inventing one based on cost-effective widespread technology such as qPCR and ddPCR.

## 2 Gliomas molecular markers

**FIGURE 1:**
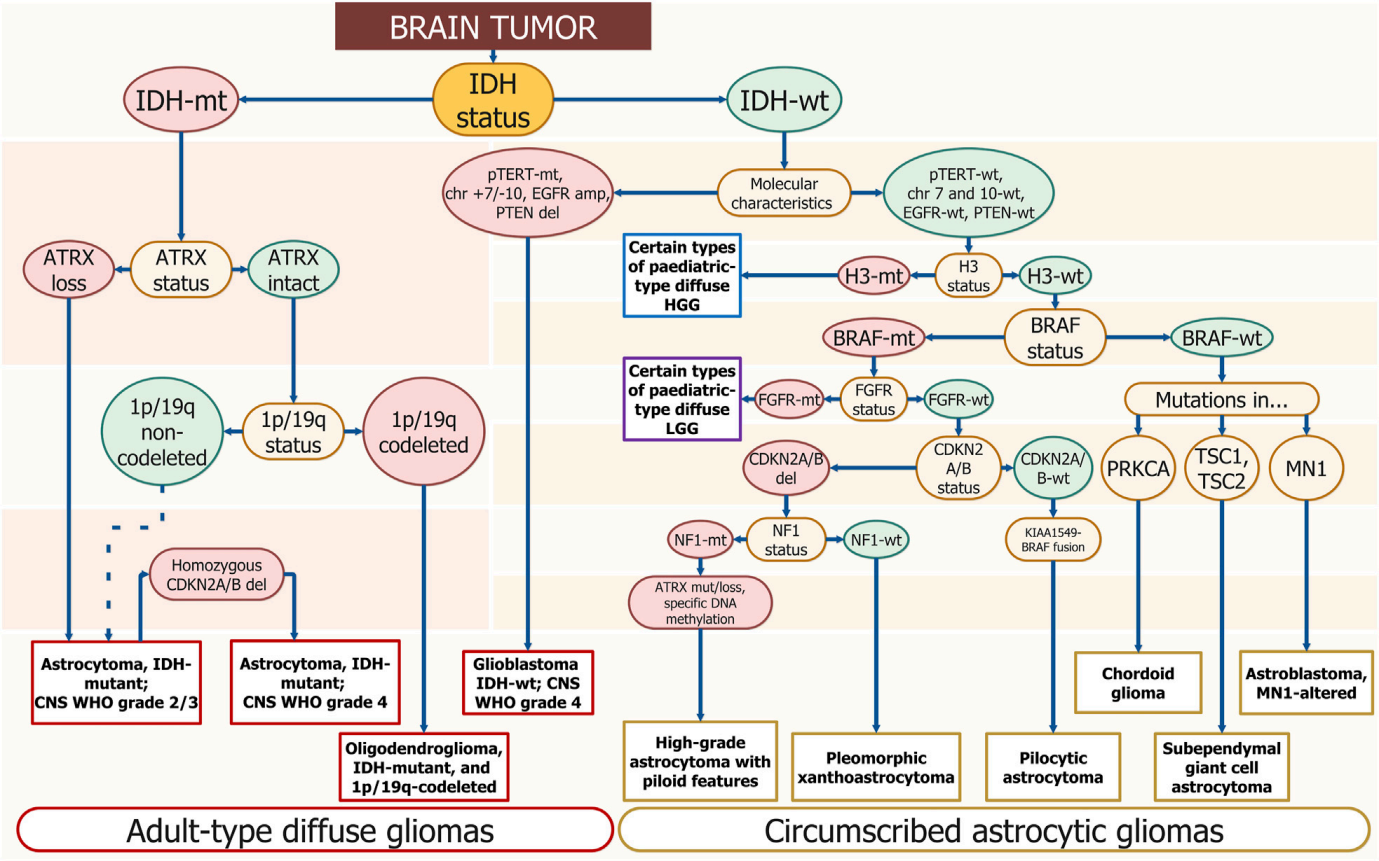
Schematic diagnostics path for diffuse glioma by gene/genomic alterations. The DNA sequence alterations are placed step by step for gradual diagnosis specification. The enquired DNA sequence alterations are annotated in yellow ellipses. Wild-type genotypes are placed in green ellipses and mutants are placed in red ones. The corresponding final diagnosis is placed in white rectangles; long ovals combine general diagnostic families — “Adult-type diffuse gliomas” and “Circumscribed astrocytic gliomas.” Resembling algorithms were demonstrated in other studies but using different approaches and covering smaller amounts of diagnosis. The suggested scheme uses only molecular profiling to determine the type of tumor (Weller et al., 2021; Brat et al., 2022).

Pathogenic DNA sequence alterations in glioma influence cell fate and the ability to evade standard treatment (Sharma, 2009). Adult-type diffuse gliomas are defined by the presence of pathogenic DNA sequence alterations in the isocytratedehidrogenese-1/2 gene (*IDH1/2*) (Sharma, 2009). They can be subclassified as either astrocytoma or oligodendroglioma by the presence of the tumor protein p53 (*TP53*) pathogenic alterations and alpha-thalassemia/intellectual disability X-linked syndrome (*ATRX*) loss in the first case, or by the revealing complete deletion of both of the short arm of chromosome 1 (*1p*) and the long arm of chromosome 19 (*19q*) (*1p/19q-co-deletion*) or telomerase reverse transcriptase (*TERT*) promoter (*p-TERT*) pathogenic DNA sequence alterations in the second (Figure 1). *p-TERT* pathogenic DNA sequence variants exclude the possibility of *ATRX* loss, as they both lead to the same consequence of telomerase activation within the tumor cell. Mutations in *IDH1/2* are associated with greater patient survival, however, *TP53* pathogenic variants occurring in *IDH*-mutant (*IDH1*-MT) tumors drop the survival rate significantly (Murnyak and Huang, 2021; Gulaia et al., 2022). The prognosis associated with *p-TERT* mutations is bivalent as patients carrying *IDH*-MT, *1p/19q-*codeletion, and promoter methylation of the O6-methylguanine-DNA methyltransferase (*p-MGMT*) favor in survival terms from the *p-TERT* co-occurrence (Arita et al., 2020; Vuong et al., 2020). Patients carrying wild-type (WT) *IDH* and mutant (MT) *p-TERT* have the poorest survival rate (Vuong et al., 2020).

Glioblastoma sequence alterations markers typically include *p-TERT* mutations, loss of cyclin-dependent kinase inhibitor 2A/B (*CDKN2A/B*) and phosphatase and tensin homolog (*PTEN*), amplification or truncation of epidermal growth factor receptor (*EGFR*), *TP53* pathogenic variants, as well as large chromosomal aberrations — 10 chromosome loss, 7 chromosome gain (*chr +7/-10*) (Figure 1).

According to the WHO 2021 classification, there is a wide range of molecular markers for the diagnosis of glioma that can be distinguished (Louis et al., 2021). Based on this classification we chose several DNA sequence alterations, such as *IDH1/2*, H3-3A, *ATRX*, *CDKN2A/B*, *1p/19q-*codeletion, *TERT*, MGMT*, EGFR*, that can be used as targets for diagnosis and evaluation of therapeutic response, as well as potential targets for successful treatment of glioma (Śledzińska et al., 2021; Miller, 2022; Yang et al., 2022).

### 2.1 IDH

*IDH* catalyzes the oxidative decarboxylation of isocitrate to 2-oxoglutarate. *IDH1* is encoded by the *IDH1* gene and is a part of the isocitrate dehydrogenase isoenzymes, with the other two being *IDH2* and *IDH3*. *IDH1* protein is located in the cytoplasm, peroxisome, and endoplasmic reticulum, whereas *IDH2* is located in the mitochondrial matrix (Bleeker et al., 2010). The functions of *IDH1* and *IDH2* relate to cellular defense against oxidative stress, cellular metabolism in lipid synthesis, oxidative respiration, and oxygen-sensing signal transduction (Reitman and Yan, 2010).

*IDH* mutations represent an early event in gliomagenesis and may occur combined with TP53 pathogenic variants and *1p/19q* loss (Figure 1). Most *IDH1* DNA sequence alterations in gliomas occur as a single amino acid substitution with the hot spot at the arginine at codon 132 — *R132H* and cDNA coordinates—c.395G>A (Van Den Bent et al., 2016).

Low-grade gliomas (LGGs) with IDH-MT and *1p/19q*-codeletion have the most favorable clinical outcomes and are predictive of a positive response to alkylating chemotherapy (Weller et al., 2012). The combination of *IDH1* sequence variants and *p-MGMT* methylation status in malignant gliomas extends the survival rate more than either of the pathogenic variants (Śledzińska et al., 2021).

### 2.2 *1p/19q*-codeletion

*1p/19q*-codeletion is a chromosomal alteration that is described in oligodendrogliomas (Figure 1). It is a complete deletion of the short arm of chromosome 1 (*1p*) and the long arm of chromosome 19 (*19q*) (Brandner et al., 2022). Loss of *1p* and *19q* is an early event in oligodendroglioma tumorigenesis (Pinkham et al., 2015) and could be a result of an unbalanced whole-arm translocation between chromosomes 1 and 19 with the loss of the resulting hybrid chromosome (Griffin et al., 2006; Jenkins et al., 2006). Detection of *1p/19q* loss allows for identifying tumor type more accurately than routine histological evaluation (The Cancer Genome Atlas Research Network, 2015).

*1p/19q*-codeletion allows the prediction of the best treatment response within *IDH*-MT tumors to temozolomide (TMZ) (Kaloshi et al., 2007). *1p/19q*-codeletion is a predictor of prolonged survival in patients receiving PCV (combination of procarbazine, lomustine, and vincristine) chemotherapy followed by radiotherapy compared to radiotherapy alone (Weller et al., 2012).

### 2.3 H3-3A

*H3.3* is a conserved histone protein, which is expressed outside the cell cycle and found at transcription start sites and in telomeric regions (Talbert and Henikoff, 2010; Armache et al., 2020). The *H3.3* histone is encoded by two different genes, *H3-3A* and *H3-3B*, which are located at *1q42.12* and *17q25.1,* respectively. Point mutations in *H3-3A* are associated with an epigenetic subgroup of high-grade gliomas (HGGs) and significantly worse outcomes (Khuong-Quang et al., 2012; Brat et al., 2018; Aldera and Govender, 2022).

The most common *H3-3A* pathogenic DNA sequence alteration is a single amino acid substitution at the positions *K27* or *G34* (Sturm et al., 2014; Lim et al., 2021). *H3 K27* alterations suppress the polycomb repressive complex 2 involved in epigenetic gene silencing; the tumorigenic mechanism of *H3.3 G34* has not yet been determined (Lim et al., 2021). Detection of an *H3 K27* or *H3 G34* mutation in a diffuse glioma, irrespective of histological grade, indicates high-grade biology (Korshunov et al., 2016; Brat et al., 2018).

Missense mutations in genes encoding histone *H3.3* isoforms occur in 50% of pediatric HGGs (Wang L. et al., 2022). A strong link was shown between *H3-3A* alterations and the age of HGG diagnosis. More often, the *H3-3A K27* mutation occurs in children and the *H3-3A* G34 mutation occurs in adolescents and young adults (Sturm et al., 2012; Wirsching and Weller, 2016). The WHO CNS5 classified tumors with alterations in *H3-3A* into a separate group, the pediatric type diffuse HGGs (Figure 1).

### 2.4 ATRX

*ATRX* is a gene located on the X-chromosome that encodes for an ATP-dependent helicase and provides nucleosome assembly, telomere maintenance, and histone *H3.3* deposition in transcriptionally silent genomic regions. There is a link between *ATRX* pathogenic DNA sequence alteration and a non-telomerase-dependent telomere lengthening mechanism called alternative lengthening of telomeres (ALT) promoting cellular immortality. Loss of *ATRX* results in genome instability, DNA damage, and global epigenetic dysregulation (Napier et al., 2015; Danussi et al., 2018). It is well known that loss of *ATRX* expression is significantly associated with *IDH*-MT (Ebrahimi et al., 2016; Chatterjee et al., 2018). However, the *ATRX* pathogenic variant is almost mutually exclusive with *1p/19q*-codeletion (Jiao et al., 2012) separating astrocytoma and oligodendroglioma (Figure 1).

It has been shown that *ATRX* pathogenic DNA sequence alteration correlates with glioma younger patient age, tumor histological features, and prognosis (Haase et al., 2018), as its mutations are frequently found in high-risk patients with a poor prognosis (van Gerven et al., 2022).

### 2.5 TERT

*TERT* is a rate-limiting enzyme for telomerase. DNA sequence alterations in *TERT* lead to the generation of two promoter-binding domains with the same structure, resulting in a multiplying of transcription activity. *TERT* reversely catalyzes telomerase production and maintains telomere ends by the addition of repeated sequences to the end of chromosomes (Huang et al., 2015).

The most common mutations are *C228T* (75%) and *C250T* (25%) (Mosrati et al., 2015). *TERT* promoter mutation generates identical 11 bp sequences that form a new binding site for the E26 transformation-specific transcription factor GA-binding protein, which selectively binds to the site and activates *TERT* (Chiba et al., 2017). Gliomas with the *p-TERT* mutation have shorter telomere lengths compared to gliomas without them, but at the same time expression of *TERT* is elevated (Arita et al., 2013; Heidenreich et al., 2015). *p-TERT* pathogenic variants are reported in 51% of all glioma types (Olympios et al., 2021) and together with *IDH*-WT constitute the most common genotype in glioblastoma (Figure 1) (Arita et al., 2016).

### 2.6 MGMT

*MGMT* is involved in cellular defense against mutagenesis and toxicity by cross-linking of double-stranded DNA by alkylating agents. The *MGMT* gene consists of five exons, and it is located on chromosome 10q26. The CpG-rich island covers a significant part of the *MGMT* promoter region including Exon 1. A minimal promoter and an enhancer region are located within the CpG island. In non-cancerous tissue, most CpG sites within the island are unmethylated. In tumors, the cytosines in CpG sites often carry a methyl group. These circumstances increase the affinity of proteins such as methyl-CpG-binding protein 2 and methyl-CpG-binding domain protein 2 to the DNA, which subsequently alters the chromatin structure and prevents binding of transcription factors leading to *MGMT* expression silencing (Nakagawachi et al., 2003).

Methylation of the gene promoter is associated with glioblastoma (Hosoya et al., 2022). Loss of chr 10q may also cause *MGMT* inactivation. Patients whose tumors show *p-MGMT* hypermethylation have longer overall survival (OS) from a combination of radiotherapy and TMZ (Stupp et al., 2005; Weller et al., 2012; Richard et al., 2020).

### 2.7 CDKN2A

The *CDKN2A* gene encodes multiple tumor suppressor 1 (*MTS1*) (Chen et al., 2021). The proteins that are encoded by *CDKN2A* (p16INK4A and p14ARF) and *CDKN2B* (p15INK4B) regulate the cell cycle and apoptosis and thus play the role of the tumor suppressor (Tesileanu et al., 2022). The homozygous *CDKN2A* deletion is a strongly unfavorable prognostic factor for survival outcomes of patients with *IDH*-MT or WT glioma (Lu et al., 2020; Varn et al., 2022). In diffuse *IDH*-MT astrocytoma, *CDKN2A* is a marker of the highest malignancy grade.

### 2.8 EGFR

*EGFR* (*HER1* or *ErbB1*) is a transmembrane tyrosine kinase located on chromosome band *7p12. EGFR* has a central role in cell division, migration, adhesion, differentiation, and apoptosis and is considered the most prominent oncogenes in *IDH*-WT glioblastomas (Figure 1). There can be various types of genetic alterations of *EGFR* and include deletions, overexpression, and focal amplifications (Li et al., 2016).

The most common pathogenic DNA sequence alteration is amplification, when the *EGFR* gene locus is amplified to extremely high copy number levels (Turner et al., 2017; French et al., 2019), causing protein overexpression and subsequent secondary mutations, including intragenic deletions, point mutations, and gene fusions, which lead to intertumoral heterogeneity (Lee et al., 2006). One of the amplification mechanisms is the “double minute”: extrachromosomal circular DNA fragments.

Another critical DNA sequence alteration is the deletion of 267 amino acids in the extracellular domain referred to as *EGFR*vIII (Gan et al., 2009) with subsequent loss of ability to bind canonical ligands. *EGFR*vIII is overexpressed in 50%–60% of *EGFR*-amplified glioblastomas (Heimberger et al., 2005) and constitutes a negative prognostic factor (Bonavia et al., 2012; Feng et al., 2014). *EGFR*vIII overexpression in the presence of *EGFR* amplification is the strongest indicator of a poor prognosis (Śledzińska et al., 2021).

### 2.9 PTEN

*PTEN* is a negative regulator of the phosphatidylinositol-3-kinase (*PI3K*)*/AKT* signaling pathway and is located on chromosome *10q23* (Chen et al., 2018). *PTEN* also promotes the stability and transcriptional activity of the *p53* in the nucleus and plays roles in chromosome stability, DNA repair, and cell cycle regulation. DNA sequence alterations in *PTEN* occur in dysplastic cerebellar gangliocytoma and glioblastoma (Benitez et al., 2017; Louis et al., 2021). *PTEN* loss is a marker of an unfavorable prognosis, induces macrophage M2 polarization, and promotes glioblastoma metastasis, growth, and angiogenesis (Ni et al., 2022).

### 2.10 Chromosome 7 gain and chromosome 10 Loss

*chr +7/-10* are specific molecular alterations frequently observed in adult *IDH*-WT glioblastoma (Stichel et al., 2018; Vuong et al., 2019). This genetic aberration also leads to monoallelic loss of PTEN (*chr10*) or to EGFR amplification (*chr7*).

Thus, glioma markers vary from SNPs in metabolic enzymes to large chromosomal aberrations, with each marker determining the cancer cell fate decisions, as most mutually exclusive pathogenic variants determine histological glioma subtypes. Other molecular markers are also important for revealing tumor type. Following the WHO 2021 classification, a new diagnostic approach could be developed.

## 3 Existing methods for determining the glioma molecular profile

**FIGURE 2:**
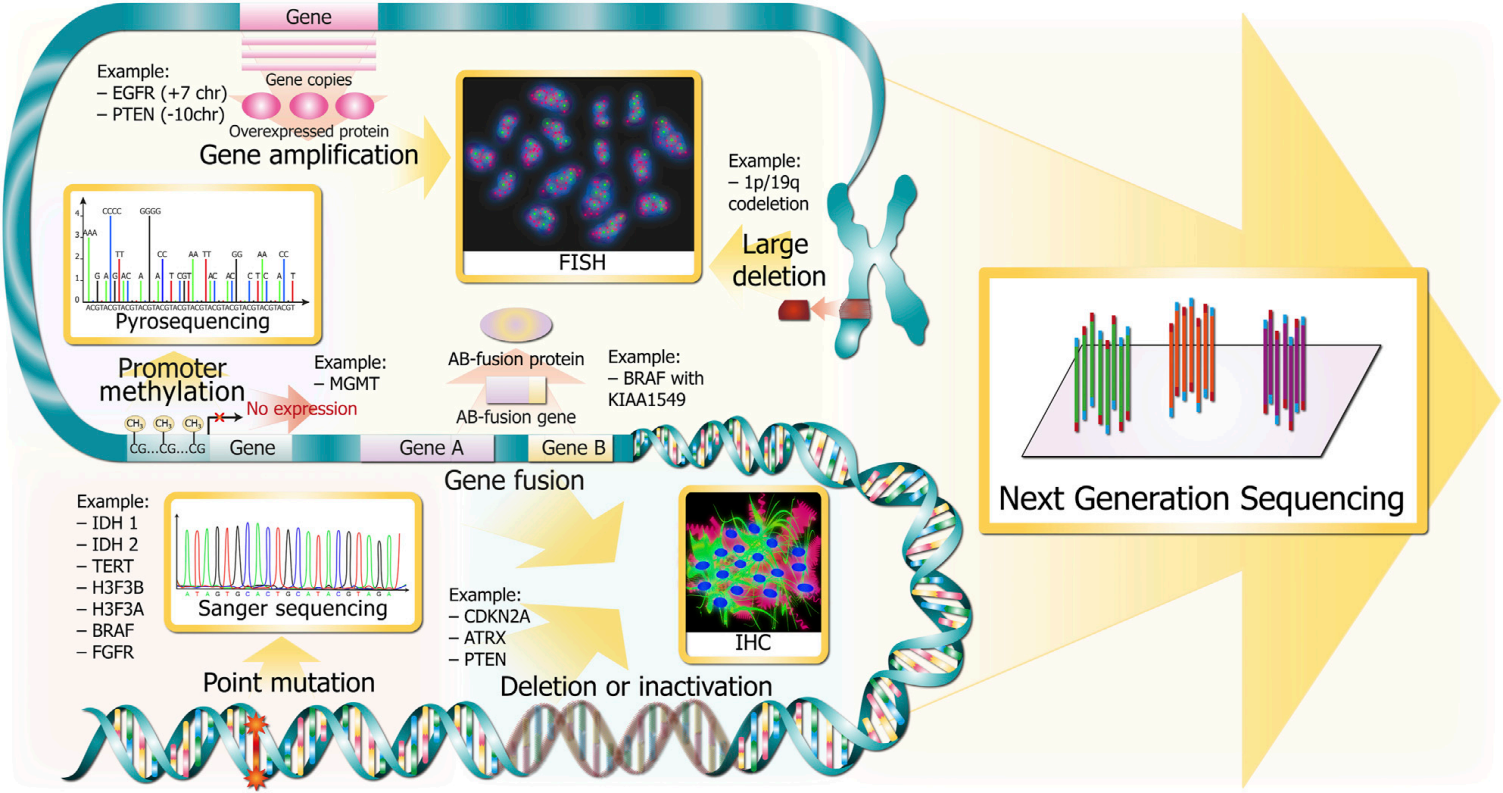
Glioma DNA sequence alterations and respective gold standards for clinical detection. Alterations are depicted in red and pink colors throughout the DNA molecule at several resolutions (from chromosomes in the upper part to single bases in the bottom). The methods for revealing the corresponding marker glioma DNA sequence alterations are depicted in golden squares and are linked with wide golden arrows.

Molecular markers of gliomas include not only point mutations in genes *IDH1/2*, *TERT*, *BRAF* (B-Raf proto-oncogene, serine/threonine kinase), but also gene deletion (*CDKN2A/B*), fusion (*BRAF* with *KIAA1549*), amplification (*EGFR*), methylation (*MGMT*), and large chromosome deletion (*1p/19q* co-deletion) (Figure 2). There are “gold standard” methods for adult glioma molecular diagnostics, namely, immunohistochemistry (IHC), fluorescence *in situ* hybridization (FISH), and Sanger sequencing. In addition to these methods, there are alternative technologies that offer numerous potential benefits: microarray, variants of PCR, NGS, optical genome mapping, and MassArray.

### 3.1 Immunohistochemistry

Immunohistochemistry is an affordable and widely available technology that was first described by Coons et al., in 1941 (Jaiswal, 2016). This method provides diagnostic of the most significant molecular markers—*IDH1* R132H, *ATRX* loss, p53 aggregation in nuclei, *EGFR*vIII, *PARP1* expression, Ki-67, *EGFR* overexpression, *BRAF* V600E, *CDKN2A*/*B* loss, *CIC* and *FUBP1* loss (Figure 2), *MGMT* positive expression (not used in clinic because of difficult interpretation and variability (Sahara et al., 2021)), and *H3-3A* K27M (Tanboon et al., 2016). *1p/19q* co-deletion is not detectable by IHC but it is mutually exclusive to *ATRX* and *TP53* pathogenic DNA sequence alterations in *IDH1*-MT gliomas (The Cancer Genome Atlas Research Network, 2015). Furthermore, there are new perspective glioma markers found recently using IHC—*BCL7A* (Liu et al., 2021) and *SRSF1* (Broggi et al., 2021) correlate with favorable prognosis, whereas *TXNDC11* (Peng et al., 2021) predicts unfavorable prognosis.

According to different studies, the IHC sensitivity and specificity for detecting various markers are close to 80%–100% (Broggi et al., 2021). The utility of IHC in detecting *IDH* DNA sequence alterations was broadened with the new antibodies to find *IDH1* R132S (SMab-1), *IDH1* R132G (GMab-1), *IDH2* R172K, *IDH2* R172M, *IDH2* R172W (KMab-1, MMab-1, WMab-1), and multispecific *IDH1/2* monoclonal antibody—MsMab-1 detecting *IDH1* R132 H/S/G and *IDH2* R172M proteins (Babakoohi et al., 2017).

### 3.2 FISH

FISH is used for target hybridization of nuclear DNA to detect alterations and gene rearrangements in paraffin-embedded tissues. This technique allows pathologists to assess such significant biomarkers as *1p/19q*-codeletion, *EGFR* amplification, *PTEN* deletion, *CDKN2A/2B* deletion (Figure 2), *BRAF* V600E, and *NTRK* fusions (Horbinski et al., 2011). There is a new FISH application, G-FISH (graphene oxide quenching-based method), that is an original technique for the detection of various RNAs (lncRNAs, miRNAs, and mRNAs) in formalin-fixed paraffin-embedded (FFPE) tissue, frozen tissue, or living cells (Hwang et al., 2019). It is used for assessing miR-21 expression as a glioblastoma prognostic marker. FISH has become a routine procedure in testing *1p/19q* codeletion because it allows analysis of paraffin-embedded tissues, does not require references and controls, and is easy to interpret. The main disadvantages include the price of probes and the necessity to establish a special dark lab. The alternative method, namely, chromogenic *in situ* hybridization (CISH), was proposed in 2013 (Lass et al., 2013) for the detection of *1p/19q* loss. According to the authors, CISH is a highly sensitive and low-cost method requiring merely a light microscope. However, data on the widespread usage of CISH in medical laboratories were not found. FISH could be used only for six markers and does not allow detection of the *IDH1* R132H pathogenic variant, which is essential in diagnostics. As a result, FISH must be used in common with IHC, NGS, or other methods.

There are cutting-edge imaging genomics methods referred to as OligoSTORM and OligoDNA-PAINT (Beliveau et al., 2017). Stochastic Optical Reconstruction Microscopy (STORM) and DNA-based Point Accumulation for Imaging in Nanoscale Topography (DNA-PAINT) enable various strategies of specific hybridization of fluorescent probes with oligos, which are used for switching individual fluorophores between dark and fluorescent states, or binding and unbinding individual labeled molecules to avoid overlapping images of single molecules. The usage of these technologies allows the resolution below 10 nm. This feature could be used in the diagnosis of glioma in creating an assay for *1p/19q*-codeletion, *chr +7/-10*, and *9p21* deletion in one reaction.

### 3.3 Sanger sequencing

Sanger sequencing was the first method to determine the DNA sequence. It has been the “gold standard” since 1977. Sanger sequencing could be used to spot various types of DNA sequence alterations, for example, SNPs and small indels. Despite the development of new sequencing technologies, such as next-generation sequencing (NGS), the Sanger method is still necessary as it produces DNA reads >500 bp with an accuracy of approximately 99.99% (Shendure and Ji, 2008). For gliomas, it is employed for the detection of important missense mutations such as *IDH1* R132, *IDH2* R172, *BRAF* V600, *TP53* R175/R248/R273, etc., (Figure 2).

### 3.4 Microarray

Microarray is a method that allows measuring gene expression from the complete genome in a single experiment. This technology is based on the specific hybridization of sample mRNA linked to fluorescent dyes with the spotted DNA sequences on the microarray. The intensity of fluorescence correlates with a gene expression and could be detected by a special laser (Tao et al., 2017). The variation of this technology is a comparative genomic hybridization (array-CGH), which enables the analysis of chromosomal copy-number variations (CNVs) (Ruano et al., 2010). Microarray could be used to predict the glioma’s response to therapy, patient survival, and treatment outcomes according to the determination of upregulated and downregulated genes (Tao et al., 2017). There is a panel for glioma molecular diagnostics that detects DNA sequence alterations to find the cancer-associated copy-neutral loss of heterozygosity (Oncology Microarray - Targeted Gene and Region Panel, 2023). Chromosome microarray provides detection of 1p/19q-co-deletions, *chr +7/-10*, deletion of *CDKN2A/2B*, *PTEN*, and loss of heterozygosity (LOH) of *17q* (Buckner et al., 2017).

### 3.5 PCR or qPCR

#### 3.5.1 High resolution melting

High resolution melting (HRM) is based on the melting temperature difference of small DNA fragments following PCR and is used to detect SNPs. There have been several applications for glioma molecular diagnosis, for instance, the ones based on the temperature difference between WT and MT fragments: IDH1/2 and *BRAF* diagnostics (Hatae et al., 2016), *TP53* assessment (Saito et al., 2021), *H3-3A*, and *H3-3B* (Yokogami et al., 2018). Other approaches increase the amount of MT allele and combine conventional PCR with COLD-PCR (co-amplification on low denaturation temperatures) for IDH1 R132H (Boisselier et al., 2010), as they are based on the statement that Tm of MT-WT heterodimers would be lower than Tm of MT-MT or WT-WT homodimers. There is a methylation-sensitive high resolution melting (MS-HRM) that is used for *p-MGMT* methylation analysis (Switzeny et al., 2016; Majchrzak-Celińska et al., 2020). However, this method has not been widely used both in clinical and scientific research since it does not allow determination of homo- or heterozygosity and is difficult to interpret.

#### 3.5.2 Allele-specific qPCR

Allele-specific qPCR is actively used in clinical oncological diagnostics to assess common pathogenic variants, especially SNPs, in a variety of cancers. It is more sensitive and rather faster than Sanger sequencing. A wide range of allele-specific qPCR approaches are known, including probe-based chemistries (TaqMan, Scorpions, Molecular Beacons), modifications of primers and probes (locked nucleic acids (LNA), and minor groove binders (MGB). TaqMan is the most popular qPCR chemistry. This method uses specific hybridization of MT and WT probes with complementary sequences on target DNA. Each oligonucleotide probe has a fluorophore and a quencher on its ends and binds either to MT or WT DNA. During PCR, a polymerase destroys the probe and releases fluorophore and quencher, and since fluorophore is distanced from quencher, fluorescence can be detected. Allele-specific qPCR with fluorescent TaqMan probes was successfully established for mutations in *IDH1/2* (Catteau et al., 2014), *H3-3A* (Zhang et al., 2016), and other point mutations.

LNA base is a type of nucleic acid analog that contains a 2′-O, 4′-C methylene bridge. This modification restricts the flexibility of the ribofuranose ring, which significantly increases the primer specificity for the target mutation. Such primer modification is well-used in TERT promoter diagnostics (Diplas et al., 2019).

A comparative quantitative PCR (CQ-PCR) was developed based on quantitative analysis of gene copies from *1p* and *19q* relative to reference genes from unaltered chromosomes (Chaturbedi et al., 2012).

#### 3.5.3 Peptide nucleic acid-mediated PCR technologies

Peptide nucleic acid-mediated PCR technologies (PNA) is an artificial DNA-oligonucleotide analog, where the phosphate-ribose backbone is replaced with a peptide-like amide backbone (N-(2-aminoethyl)glycine), which leads to increased stability of binding to DNA. Several variations of PNA application, e.g., PNAClamp, PANA RealTyper, PANA qPCR, and PANAMutyper, are united by a PNA probe hybridizing to complementary DNA sequence and precluding polymerase elongation. PNAClamp is used to specifically block the amplification of WT DNA and increase the synthesis of MT DNA. It provides accurate detection of 1% MT DNA in 50 ng of tissue and is applied to detect DNA sequence alterations in *BRAF*, *IDH1*, *IDH2*, *PIK3CA*, and *TERT* in tissue biopsy (Choi and PANAGENE, 2023). PANA RealTyper probe contains fluorophore and quencher, so the fluorescence signal is detected when the probe is annealed on target DNA, but if the PNA-DNA complex denatures the signal disappears. This PANA RealTyper is applied in the same manner as HRM analysis and is similar to PANA qPCR, whereas PANAMutyper combines both of the previously described techniques. PNA oligos are employed in diagnostic kits to assess glioma-associated pathogenic variants in liquid biopsy.

#### 3.5.4 Droplet digital PCR

Droplet digital PCR (ddPCR) is now actively used to reveal pathogenic variants in samples with very low concentrations of tumor DNA, as it allows to determine as low as 2% of MT DNA. As profiling tumor tissues from the brain could be challenging because of tumor location and small sample size, currently, researchers are trying to adapt the use of cell-free tumor DNA (ctDNA) from cerebrospinal fluid (CSF) and plasma for revealing glioma molecular markers. Taking into account that the blood-brain barrier restricts the amount of ctDNA and generally brain tumors do not grow much in size because of space limits, the identification of glioma marker DNA sequence alterations in patient plasma is technically difficult (Mair and Mouliere, 2022). CSF is a more reliable ctDNA source for glioma patients, however, a lumbar puncture could be deleterious to patients and cannot be applied frequently. The sensitivity of ddPCR detection of *p-TERT* MT in patient plasma by ddPCR was 62.5% (Muralidharan et al., 2021). The technical ddPCR variation—BEAming comprises emulsion PCR and the flow cytometry to separate WT and MT DNA as different populations. However, the sensitivity of detecting ctDNA in glioma patients was less than 10% (Bettegowda et al., 2014).

### 3.6 NGS

NGS is a fast-growing cutting-edge methodology employing little amount of sample and is applicable to detect different types of DNA sequence alterations: SNPs, small indels, amplifications, big regions of LOH, and CNAs (Figure 2). NGS could be used for all essential pathogenic variants in glioma, and it is a more sensitive and specific method than IHC (Śledzińska et al., 2022). Plenty of NGS panels have been developed for glioma molecular diagnosis: glio-DNA panel (Tirrò et al., 2022) and GliomaSCAN (Shin et al., 2020). However, there is no CE-IVD or FDA-approved NGS panel, and not every hospital is able to establish NGS facilities due to its cost, unavailability of necessary specialists, and difficulties in providing consumables.

There are several types of NGS, specifically, pyrosequencing, sequencing by ligation, sequencing by synthesis, nanopore technology, and DNBseq.

#### 3.6.1 Pyrosequencing

Pyrosequencing platform Roche 454 was the first NGS method. This platform uses emulsion PCR with the subsequent addition of a purified nucleotide, which incorporates into the DNA strand and results in the release of pyrophosphates. Then the series of enzyme reactions with pyrophosphate produces a flash of light (Jessri and Farah, 2014). The main advantages are long reads (approximately 700 bp), short run time, and high accuracy in homopolymer regions. This technology could be used to detect MGMT promoter methylation but is less effective than PCR-based alternatives due to its cost (Switzeny et al., 2016).

#### 3.6.2 Sequencing by ligation (SBL)

SBL is based on the specific hybridization of known fluorescent oligonucleotides with target sequences. This method is good for detecting SNP and gross chromosome abnormalities (Slatko et al., 2018). Despite its high specificity and low rate of errors, it is very difficult to analyze the great amount of data (over 180 GB) and hence very rarely used for commercial platforms (Life TechnologiesTM SOLiD™ 5500XL) (Jessri and Farah, 2014).

#### 3.6.3 Sequencing by synthesis

Sequencing by synthesis (SBS) is the most common NGS method for sequencing without using dideoxy terminators. Ion Torrent platforms use emulsion PCR and identification of nucleotide incorporated by the released H^+^ ion by detecting the pH change. Illumina platforms utilize bridge PCR with fluorescent nucleotides incorporated in the growing DNA strand, and the optical detector catches fluorescent signals. After fluorophores are removed, the next nucleotides can be joined. However, it produces short reads with a high error rate (Jessri and Farah, 2014).

#### 3.6.4 Nanopore

Nanopore technology is considered third-generation sequencing, as it enables real-time analysis of long strands. It measures differences between electronic changes in a special membrane, which depends on the nucleotide going through the nanopore. It does not require PCR amplification before and has a very high speed (400 bp per second) (How nanopore sequencing works, 2023). The downbreak is the comparatively low read accuracy as it produces ultra-long reads, of over 20kbp (Jain et al., 2018), and thus cannot be used for SNV detection (Kono and Arakawa, 2019).

#### 3.6.5 DNBSeq

DNBSeq is a Chinese sequencing technology. The library preparation ligates nucleic acids to make a single-stranded DNA circle, which is replicated to produce DNA nanoball read by various sequencing platforms including BGISEQ-500 (Fehlmann et al., 2016), DNBSEQ-G400, and DNBSEQ-T7. This method is used for SNV and small indel detection. Although it produces short reads (around 50 bp), it shows high-quality results and deep coverage (Jeon et al., 2021).

#### 3.6.6 CAPP-seq

Cancer Personalized Profiling by Deep Sequencing (CAPP-seq) is a personalized method for monitoring the tumor evolution in a patient without invasive procedures. In the first step, the molecular profile of the tumor is determined in tissue using NGS producing a unique specific library. The next step for monitoring disease relapse is to collect blood from the patient to search for circulating tumor DNA (ctDNA). CAPP-seq uses oligonucleotide probes with biotin specific to frequently mutated marker genes in a tumor. After hybridization with these probes, target DNA is cached by streptavidin sticking to biotin. However, CAPP-seq is unlikely to be applied for monitoring patients with glioma, as ctDNA is detected at extremely low concentrations in the blood due to the blood-CSF barrier (Newman et al., 2014; 2016).

### 3.7 Optical genome mapping

Optical genome mapping (OGM) is a new method for high-resolution detection of genomic structural variants, including loss, multiplication, rearrangement, and translocation of large genomic regions (Dremsek et al., 2021). DNA is labeled with the unique barcodes detected while DNA is moving through the nanochannel. Subsequently, the results are compared with the reference barcode pattern. OGM analyzes linearized DNA strands 200 kbp in length and is applicable to create maps covering the whole chromosome arm to detect indels larger than 500 bp, translocations larger than 50 kb, inversions larger than 30 kbp, duplications larger than 30 kb, and CNVs larger than 500 kb (Bionano, 2023). The limitations relate to the detection of aneuploidies, Robertsonian translocations, and other whole-arm translocations involving centromeres (Dremsek et al., 2021). OGM could become a prospective alternative to FISH.

### 3.8 Padlock probe-based amplification

A padlock probe is a linear oligonucleotide with two target complementary arms joined together by template-dependent ligation. After the ligation, there is a rolling circle amplification step leading to the formation of 1-μm-sized coiled structures. These structures could be visualized by FISH. This molecular analysis is performed directly in fixed cells or tissue (Nilsson et al., 1994; Larsson et al., 2004). There are several modifications of the padlock probe, for example, gap-fill ligation to assess SNPs *in situ* (Mignardi et al., 2015). In this method, target complementary arms form a 6-nucleotide gap. Then MT and WT probes are hybridized with the gap, but one of these two probes (e.g., WT) blocks rolling-circle amplification (RCA). As a result, only one variant (e.g., MT) is amplified. Padlock probes can be adapted for diagnostic fusion mutations (Chen et al., 2022). The base-specific *in situ* sequencing (BaSISS) method utilizes padlock probes with unique barcodes (Lomakin et al., 2022). The development of this methodology fosters multiplexing detection of SNP, CNA, and expression analysis, which can be employed in clinical diagnostics.

### 3.9 MassARRAY

MassARRAY genotyping is a simple, high-quality, cost-effective, and comparatively fast (∼8.5 h) method providing simultaneous detection of SNPs, CNVs, indels, and methylation analysis. This technology is based on matrix-assisted laser desorption/ionization-time of flight (MALDI-TOF) mass spectrometry. The procedure includes DNA extraction, PCR, and single base extension reaction with primer, which anneals directly before the hot spot polymorphic base and ‘terminator’ nucleotide (Ellis and Ong, 2017). After that, the mixture is transferred to the SpectroCHIP, which is placed in the MassARRAY mass spectrometer (The MassARRAY System from Agena Bioscience, 2023). The determination of the polymorphic base is carried out according to the difference in molecular weight of each nucleotide. This method allows accurate detection of up to 40 SNPs in one tube and could be performed in 24-, 96- or 384-well plates (The MassARRAY System from Agena Bioscience, 2023). The MassARRAY System is actively used both in clinical and scientific research because it does not require fluorescent detection or special rooms, can acquire many different analyses at one time, and the price of each sample is very low. There are developed panels for glioma diagnosis incorporating *1p/19q*-codeletion, *IDH1* R132, *IDH2* R172, *TERT* G228A, and G250A DNA sequence alterations (Pesenti et al., 2017). High accuracy, effectiveness, low cost, and speed make this method excellent for use in clinical laboratories. However, for now, the MassArray panel is not clinically approved.

All in all, there are a wide variety of methods for detecting glioma molecular markers because of the latter’s high genetic heterogeneity and cellular plasticity. Currently, great expectations are invested in the NGS-based methods, which allow for all pathogenic variants to be covered by one technology and possess the ability to screen dozens to hundreds of patients simultaneously. Meanwhile, there are two techniques, namely, MassARRAY and ddPCR, that promise to revolutionize the glioma molecular profile. MassARRAY even exceeds the capacity of DNA sequence alteration detection by covering the possibility to enquire about the MGMT-promoter methylation along with SNPs and CNVs in a single experiment. On the other hand, ddPCR or qPCR could be used as a sensitive method to cover the genes related to therapeutic resistance/response in gliomas—*IDH1* R132H, *IDH2* R172K, *EGFR* and *MGMT* expression. Thus, we suggest these two alternatives to NGS-based techniques.

## 4 The market for glioma molecular profile

The biopharmaceutical companies provide their solutions with kits for researchers (research use only—RUO) and clinicians with CE-IVD certificate (European approval), FDA (United States), or National Medical Product Administration (NMPA, China) approval. Here, we summarize commercial offers of RUO and clinical kits available for glioma molecular profiling worldwide.

### 4.1 PCR kits

The easiest way to detect glioma marker DNA sequence alterations is to use a wide variety of PCR strategies discussed above. With PCR, we focused on the marker glioma SNP detection illustrated in Figure 1. We discovered that eight companies offer qPCR kits to identify DNA sequence alterations in *IDH1*, *IDH2*, *TERT*, *EGFR*, *BRAF*, *PIK3CA*, and methylation of *MGMT* promoter and *EGFR*vIII; two companies provide ddPCR kits for the determination of *IDH1* R132H and *IDH2* R172K mutations, *EGFR* amplification, *CDKN2A* loss of heterozygosity, and *TERT* CNV; one company promotes PNA PCR technology to detect DNA sequence alterations in *BRAF*, *IDH1*, *IDH2*, *PIK3CA*, and *TERT*.

The determination of *IDH1/2* mutations is offered by the majority of companies and most of their kits are clinically approved either by the FDA: Abbott (United States) (RealTime IDH1 | Abbott Molecular, 2023; RealTime IDH2 | Abbott Molecular, 2023) or CE-IVD: PANAGENE (South Korea) (Choi and PANAGENE, 2023), Genetron (China) (CANCER DIAGNOSTICS, 2023), EntroGen (United States) (IDH1/2 Mutation Detection Kit | EntroGen, Inc, 2023), Qiagen (Germany) (therascreen IDH1/2 RGQ PCR Kit CE, 2023). NeoGenomics (United States) promotes a kit for RUO (IDH1/IDH2 Mutation Analysis by PCR | NeoGenomics Laboratories, 2023). All companies except Abbott allow the use of their kits for glioma molecular diagnostics with DNA extracted from FEPE tissue. According to their guidelines, qPCR kits can catch as low as 1% mutant DNA in mixed samples.

Some companies, such as PANAGENE (South Korea) (Choi and PANAGENE, 2023), Genetron (China) (CANCER DIAGNOSTICS | GENETRON, 2023), JBS Science (United States) (TERT promoter −124 mutation Quantification kit – JBS Science, 2023), market kits to detect *hTERT* promoter DNA sequence alterations C228T and C250T. PANAGENE has a CE certificate and recommends its kit for CNS tumors diagnostics, Genetron allows the use of its kit for glioma profiling and has CE-IVD, and JBL Science offers a kit to identify C228T for RUO and it could be used only on Roche’s LightCycler™.

Additionally, Genetron (China) provides an 8-gene kit comprising DNA sequence alterations in *EGFR*, *BRAF,* and *PIK3CA* (CANCER DIAGNOSTICS | GENETRON, 2023) approved for medical use by the National Medical Products Administration (“NMPA”) in China. PANAGENE (South Korea) promotes a *PIK3CA* mutation detection kit that has CE-IVD. EntroGen (United States) offers a kit to detect *EGFR*vIII amplification (EGFRvIII Detection Kit, 2023).

The methylation of *MGMT* promoter analysis is designed by three companies only: EntroGen (United States) (MGMT Methylation Detection Kit | EntroGen, Inc, 2023), NeoGenomics (United States) (MGMT Promoter Methylation Analysis | NeoGenomics Laboratories, 2023), and Genmark Sağlık Ürünleri (Turkey) (geneMAP™ MGMT Methylation Analysis Kit MGM-RT50, 2023). EntroGen and Genmark kits are CE-approved and the NeoGenomics kit is New York-approved. EntroGen (United States) declared the ability of its kit to detect as low as 6.25% methylated DNA in mixed samples.

Despite neither of the ddPCR kits being clinically approved, Bio-Rad Laboratories (United States) and IdSolutions (France) offer very sensitive kits for revealing *IDH1* R132H, *IDH2* R172K, and *TERT*-promoter mutations. Bio-Rad also detects *EGFR* amplification and *CDKN2A* loss of heterozygosity. This technology is more sensitive than Sanger sequencing (Wolter et al., 2022) and could facilitate the development of liquid biopsy for glioma diagnostics. With their declared high sensitivity (<0.1%), these kits could be applied for DNA extracted from FFPE tissue and ctDNA extracted from plasma.

In summary, nowadays there is no PCR-based kit that would provide detection of enough pathological markers in glioma. However, there are CE-approved kits to assess mutations in *IDH1/2*, *p-TERT*, methylation of *p-MGMT*, and amplification of *EGFR*vIII. Although the most advantageous kits for glioma are based on ddPCR due to their high sensitivity, they are not clinically approved and are the most expensive as they require a special PCR cycler. Therefore, companies offer solutions utilizing conventional qPCR, which is cost-effective and could combine different essential diagnostic markers.

### 4.2 FISH

There are three main manufacturers in the FISH probe market, namely, Cytocell (United Kingdom), Abbot Molecular (United States), and ZytoVision (German), which provide clinically approved FISH kits for glioma profiling (Table 1). There is a Taiwan company, Abnowa, which produces FISH probes for RUO, however, it also uses mutation-specific FISH technology to detect DNA sequence alterations (mutaFISH™ (mutaFISH™ - Technologies - Support - Abnova, 2023)).

**TABLE 1:** FISH kits for the diagnosis of gliomas.

Regions important for the diagnosis of glioma	CytoCell	Abbott molecular	ZytoVision	Abnova
1p19q codeletion	1p36.32/1q25.2	1q25.2/1p36.3	1p36/1q25	1q43-q44/1q21.3 19q13.13/19q13.11
	19p13.2/19q13.33	19q13.3/19p13.2	19q13/19p13	
EGFR amplification	7p11.2	7p11.2	7p11.2	7p12
	7p11.1-q11.1	7p11.1-q11.1		
PTEN 10q	10q23.2-10q23.3	10p23	10p23.2-q23.31	10q23/10p11.22
CDKN2A/B deletion	CDKN2A deletion 9p21.3	CDK2A deletion 9p21	CDKN2A deletion 9p21.3	**-**
	9q12			
BRAF V600E	—	—	—	BRAF V600
BRAF fusion	KIAA1549/BRAF 7q35	—	—	KIAA1549/BRAF 7q34
TP53	—	17p13.1 CNV	17p13.1	17p13.1/9p21/18q21.2
Medical diagnostic license	CE-IVD, RUO	CE-IVD	CE-IVD	RUO

For glioma molecular profiling all these companies offer probes for detection of *1p/19q*-codeletion, *EGFR* amplification, and *PTEN*. Despite missing probes for the detection of *CDKN2A* deletion, Abnova developed *BRAF* V600E diagnostic probes. *BRAF* fusion is identified with probes from Cytocell and Abnova. The determination of *TP53* CNV is provided by Abbot Molecular, ZytoVision, and Abnova.

FISH is a gold standard for the diagnosis of chromosomal aberrations. Despite it being essential in the detection of *1p/19q-*codeletion and *chr +7/-10* in glioma, FISH is expensive, labor-consuming, and limited, making it difficult to create a cost-effective FISH kit for profiling different levels of DNA sequence alterations other than chromosomal rearrangements.

### 4.3 NGS panels

There are no targeted FDA or CE-IVD-certified NGS panels for patients with glioma. This could be due to the low incidence of glioma in the general population and the impossibility of non-invasive molecular diagnostics because of the difficulties in detecting ctDNA in patients with glioma unless it is at an advanced stage. However, the multi-cancer panels offered in the medical field could detect a part of the genes needed for diagnosing gliomas. Meanwhile, there are several RUO kits featured for the profiling of patients with glioma. An additional branch of the market for glioma molecular characterization is devoted to clinically certified diagnostic laboratories providing glioma DNA sequence alterations detection by NGS as a medical service. However, we were not able to comprehensively describe the latter, because the laboratories merely address the information related to patient sample handling and do not describe either the genes covered by the service or the platform used for NGS.

There are several FDA-approved pan-cancer NGS panels, namely, MSK-IMPACT (Memorial Sloan Kettering Cancer Center, New York, United States), FoundationOne Liquid CDx (Foundation Medicine, Cambridge, United States), and Guardant360 CDx (Guardant Health, Palo Alto, United States), with the two latter approved for liquid biopsy. The panels designed for ctDNA detection use barcode enrichment to identify rare DNA sequence alterations. Both panels can detect a rare pathogenic variant with the variant allele frequency (VAF) ranging from 0.40% to 0.82% and a probability of 95% (Vasseur et al., 2022). The panels have additional benefits by detecting CNVs important for the diagnosis of glioma (Jonsson et al., 2019; Sharaf et al., 2021) except for Guradant360 CDx. However, none of the multicancer panels incorporated *MGMT*-promoter methylation (Table 2). MSK-Impact can cover 468 genes, FoundationOne CDx assay can cover 324 genes, and Guardant360 CDx can cover 360 genes. The comparison of the panel performances revealed equal quality in identifying mutational burden (Bevins et al., 2020), although the enrichment strategy is different. FoundationOne Liquid CDx and Guardant360 CDx use hybridization and capture-based enrichment utilizing oligonucleotides complementary to genes of interest, that serve as baits to capture hybridizing DNA fragments (Gaudin and Desnues, 2018). MSK-IMPACT exploits capturing beds with oligos hybridizing with target DNA fragments and amplified by ligation-mediated PCR.

**TABLE 2:** Pan-cancer NGS panels that are available in the clinical and research markets with possible implementation for glioma molecular profiling.

Genes important for glioma molecular profiling	OncoDEEP from OncoDNA	Oncomine pan-cancer cell-free assay	Tempus xT	MSK-IMPACT	FoundationOne^®^ liquid CDx	Guardant360 CDx	OncoScreen panel from burning Rock Dx	PGDx elio plasma complete
IDH1/2, BRAF, EGFR, KIT, TP53, CDKN2A, PTEN	+	+	+	+	+	+	+	+
p-TERT	-	-	+	+	+	+	-	+
H3-3A (H3F3A)	+	-	+	+	+	-	-	+
HIST1H3B	+	-	+	+	-	-	-	+
FUBP1	+	-	+	+	+	-	-	+
CIC, NOTCH1	+	-	+	+	+	-	+	+
CDKN2B, ATRX	+	+	-	+	+	-	+	+
1p/19q-codel	-	-	+	+	+	-	-	-
+7chr/-10chr	-	-	-	+	-	-	-	-
p-MGMT methylation	-	-	-	-	-	-	-	-
Platform	Illumina	Ion Torrent S5	Illumina HiSeq, NovaSeq 6000	Illumina HiSeq 2500	Illumina NovaSeq 6000	Illumina NextSeq 550	Illumina NextSeq 500	Illumina NextSeq
Biological material	FFPE tissue	ctDNA	FFPE tissue	FFPE tissue, ctDNA	FFPE tissue, ctDNA	ctDNA	FFPE tissue	ctDNA
Medical diagnostic license	CE-IVD	RUO	CE-IVD	FDA	FDA	FDA	CE-IVD	RUO

Personal Genome Diagnostics (PGDx; Baltimore, United States) and NuProbe (Houston, United States) offer liquid biopsy pan-cancer panels for RUO. Both panels include important genes for glioma, however, PGDx elioTM plasma for solid tumors is intended for 500+ genes (Valkenburg et al., 2022) and can detect most of the glioma markers including *p-TERT* DNA sequence alterations (Alnajar et al., 2022) (Table 2). The NuProbe Pan-Cancer NGS panel covers 61 genes (NuProbe Launches Liquid Biopsy Pan-Cancer NGS Panel, 2023) and uses the special enrichment technique referred to as quantitative blocker displacement amplification (QBDA) for detecting rare genetic variants below 0.01% VAF (Dai et al., 2021). This feature could be potentially used for glioma ctDNA profiling. Due to the enrichment technique, the panel can be run with low-coverage sequencers, such as Illumina MiSeq and MiniSeq, which makes it cost-effective compared to other panels of the same size.

Tempus (Chicago, United States) offers a CE-certified NGS panel, Tempus xT, to search for DNA sequence alterations in 648 genes, including almost all important genes in glioma (Table 2), and could be used to detect *1p/19q*-codeletion (Beaubier et al., 2019) in patient tumor material (FFPE tissues). Another option is the clinical Tempus xF panel that is to be used for molecular profiling of ctDNA and covers 105 genes (Tempus xF - Clinical test - NIH Genetic Testing Registry GTR - NCBI, 2023) including glioma marker DNA sequence alterations except of ATRX, CIC, FUBP1, and TERT-promoter (Genomic Profiling - Tempus, 2023).

Illumina (San Diego, United States) sells TruSight Oncology 500/500 ctDNA RUO panel investigating 523 genes in tumor specimen or ctDNA (Wei et al., 2022) covering all glioma marker SNPs including p-TERT DNA sequence alterations (Kang et al., 2022). The possibility of identifying CNVs, such as *1p/19q* codeletion, for the diagnosis of glioma was proven only for the TruSight Tumor 170 panel (Na et al., 2019), which, however, lacks the important glioma diagnostic genes *ATRX*, *CIC*, *FUBP1*, and *CDKN2B*. EU market trades TruSight Oncology Comprehensive examining 517 genes under CE-IVD certificate (TruSight Oncology Comprehensive EU, 2023). Myriad Genetics (Salt Lake City, United States) features the RUO NGS panel, Precise™ Tumor, for solid tumor characterization covering 523 genes by DNA sequencing and with most glioma-related SNPs, but missing *p-TERT* DNA sequence alterations (Precise™ Tumor | Molecular Profile Testing | Myriad Genetics, 2023).

Thermo Fisher Scientific (Waltham, United States) offers several NGS kits for cancer diagnostics—Oncomine Focus with 52 genes for solid tumors (Oncomine Focus Assay | Thermo Fisher Scientific, 2023), Oncomine Comprehensive Assay v3 with 161 genes, and Oncomine Comprehensive Assay Plus including 501 genes (Vestergaard et al., 2021), with the latter having the possibility to identify 1p/19q-codeletion (Ali et al., 2023). Additionally, the Oncomine Pan-Cancer Cell-Free Assay designed for ctDNA profiling detected 67% of DNA sequence alterations found in the corresponding primary tumor in CSF ctDNA from patients with CNS malignancies (Shah et al., 2021). Another option is the Ion AmpliSeq Cancer Hotspot panel, an assay covering 50 genes, that was investigated for the substitution of gold-standard techniques for tumor profiling (Lee et al., 2018). The panel has drawbacks for patients with glioma as it can be used only for the genomic DNA from tumor tissue and misses a lot of clinically important genes. All described Thermo Fisher NGS panels are RUO.

Mayo Clinic Laboratories (Rochester, United States) offers a MayoComplete Solid Tumor panel investigating SNVs and indels within 515 genes in the FFPE tissues (MCSTP - Overview: MayoComplete Solid Tumor Panel, Next-Generation Sequencing, Tumor, 2023). The panel can be used for profiling patients with glioma as it includes most of the essential glioma SNVs, missing only *p-TERT* DNA sequence alterations. There is a neuro-oncology panel (NONCP) covering 118 genes from tissue blocks. The panel could be used for profiling pediatric gliomas, but it misses the important diagnostic CNVs (NONCP - Overview: Neuro-Oncology Expanded Gene Panel with Rearrangement, Tumor, 2023). The Jackson Laboratory (Sacramento, United States) offers the JAX SOMASEQ NGS panel covering 517 genes for profiling patient DNA from FFPE tissues. It is compatible with Illumina Novaseq 6000 (JAX SOMASEQ - Clinical test - NIH Genetic Testing Registry GTR - NCBI, 2023) and could be used for the diagnosis of glioma, however, it misses *p-TERT* DNA sequence alterations, *p-MGMT* methylation, and important CNVs such as *1p/19q* codeletion. Paragon Genomics (Hayward, United States) offers the RUO CleanPlex OncoZoom Cancer Hotspot kit that examines 65 pro oncogenes and antioncogenes compatible with Illumina MiSeq and IonTorrent platforms (Zhu et al., 2021; CleanPlex OncoZoom Cancer Hotspot Kit for Cancer Profiling, 2023). CellMax Life (Sunnyvale, United States) developed CellMax-LBx to detect DNA sequence alterations in 73 genes in the ctDNA of various patients with cancer (Liu and Wang, 2022). So far, it lacks a medical certification but includes important glioma molecular markers, such as pathogenic DNA sequence alterations in *IDH1*, *IDH2*, *BRAF*, *CDKN2A*, *PTEN*, *NOTCH1*, and *TP53*.

Natera (Austin, United States) offers the Signatera NGS kit that is specially designed for ctDNA profiling and could be customized for a particular patient. Sequencing on corresponding tumor tissue and identification of the molecular markers might be used to search the patient’s blood during relapse monitoring. Thus, there is a possibility to design a molecular residual disease monitoring system for each patient with glioma (Loupakis et al., 2021). Additionally, Altera is designed to profile 440 medically important genes by whole genome sequencing (WGS) including all glioma-relevant SNPs except for *p-TERT* DNA sequence alterations (Comprehensive Genomic Profiling, 2023). CD Genomics (New York, United States) features targeted NGS Glioma Gene Panel (Glioma Gene Panel Sequencing - CD Genomics, 2023) exploring 12 glioma-related genes in FFPE-tissues. The main disadvantages are the impossibility of *1p/19q*-codeletion detection, the large amount of DNA required (minimum 200 ng), and the compatibility only with the Illumina Hiseq platform. NeoGenomics Laboratories (Fort Myers, United States) provides an NGS service for detecting CNS tumor associated DNA sequence alterations in the FFPE tissue blocks, the NeoTYPE^®^ Brain Tumor Profile (NeoTYPE^®^ DNA & RNA - Brain | NeoGenomics Laboratories, 2023). It can be used for the detection of all essential glioma DNA sequence alterations plus *p-MGMT* methylation and *1p/19q*-codeletion. ArcherDx/Invitae (Boulder, United States) offers RUO LiquidPlex panel compatible with Illumina and detecting DNA sequence alterations in *IDH1*, *IDH2*, *BRAF*, *EGFR* and *TP53* (LiquidPlex ctDNA panel, 2023). The kit has the advantage of enrichment for rare ctDNA by amplifying highly fragmented DNA pieces typically sourced from tumors in contrast to long genomic DNA from white blood cells (Vendrell et al., 2020). A relatively low-cost solution could be the NGS Targeted Hotspot panel (CE-IVD certified) from EntroGen (Woodland Hills, United States), which covers 16 genes with several important genes for glioma (Śledzińska et al., 2022), allowing to profile up to 40 patient samples using Illumina MiniSeq or MiSeq (NGS Targeted Hotspot Panel 16 Kit | EntroGen, Inc, 2023). Personalis (Menlo Park, United States) offers a panel with 267 genes for solid tumor molecular profiling including most of the glioma-associated genes, but missing oligodendroglioma markers, namely, *FUBBP1*, *1p/19q*-codeletion, and *p-TERT* (Personalis | NeXT Dx, 2023).

Genes2Me Pvt. Ltd. (Gurgaon, India) introduced their own PanCan panel comprising oncological profiling of 524 genes (Next Generation Sequencing NGS Clinical Exome Sequencing Panels Assays | HLA Typing, 2023). However, we did not manage to find a comprehensive description of the genes used.

There are several NGS pan-cancer panels developed in China. Burning Rock Biotech produces CE-certificated panels, namely, OncoScreen spanning 295 genes including the one important for glioma profiling (Table 2) and OncoScreen Plus with 520 genes, both compatible with the Illumina platform (Tang et al., 2020). Onco PanScan (Genetron Health) is CE-approved and was designed to detect 309 microsatellite instability regions (Yang et al., 2023). RUO NGS kits are produced by Berry Genomics (BeiJianAn^®^) for whole-exome sequencing (WES), Novogene (NovoPM™ 2.0), AcornMed Biotechnology, AmoyDx (AmoyDx Comprehensive Panel; AmoyDx HANDLE Classic NGS Panel), GenePlus-Suzhou (Gene + OncoGlioma). Novogene Precision Medicine 2.0 (NovoPM™ 2.0) is a comprehensive test for all solid tumors profiling 484 genes (incorporating all glioma-related genes from Table 2) (NovoPM 2.0 - Novogene, 2023) intended for both tumor specimens and ctDNA. AmoyDx Comprehensive panel is intended for both tissue and liquid biopsy and is compatible with Illumina NextSeq 500 and NovaSeq 6000. AmoyDx HANDLE Classic NGS panel covers most of the glioma-related genes (Comprehensive Panel_Amoydx, 2023; HANDLE Classic NGS Panel_Amoydx, 2023). Gene + OncoGlioma glioma gene test covers 1,021 genes and combines NGS with hybridization enrichment and pyrosequencing to analyze *MGMT* methylation status (Gene+|OncoGlioma, 2023).

ACT Genomics Co. Ltd. (Hong Kong) offers a RUO ACTOnco + kit that covers 440 genes with the glioma-related ones (*IDH1*, *IDH2*, *BRAF*, *CDKN2A*, *CDKN2B*, *CIC*, *FUBP1*, *NOTCH1*, etc.) for solid tumor molecular profiling compatible with the Ion Torrent platform (Jan et al., 2022). Geneseeq Technology Inc., the Chinese-Canadian company formerly known as Nanjing Geneseeq Technology Inc., developed GeneseeqPrime™ RUO for sequencing 425–437 solid tumor genes from biopsy or ctDNA (Geneseeq: Solid tumor genomic testing for precision oncology, 2023). The gene set includes *IDH1*, *IDH2*, *BRAF*, *CDKN2A/B*, *PTEN,* and *NOTCH1*, but misses *CIC*, *FUBP1*, *ATRX,* and *H3-3A* important for oligodendroglioma and astrocytoma diagnosis (Ye et al., 2022). Additionally, the Gliocan RUO kit is designed to sequence DNA sequence alterations in *IDH1*, *IDH2*, *ATRX*, *BRAF,* and *TP53*, along with detecting *1p/19q*-codeletion and *p-MGMT* methylation (Central Nervous System Tumors, 2023). However, it misses oligodendroglioma important pathogenic variants in *p-TERT*, *CIC*, *FUBP,* and *NOTCH1*.

Gendia (Antwerp, Belgium) offers the oncodiagnostics service for patient ctDNA screening over 50 genes, which includes several glioma-related genes, such as *IDH1*, *IDH2*, *NOTCH1*, *PTEN*, *BRAF*, *EGFR*, and *TP53* (Plessers, 2023), but there is no description of the NGS platform used for gene sequencing. Myriapod^®^ NGS OncoDNA (Gosselies, Belgium) offers a CE-IVD kit - OncoDEEP for sequencing of 638 genes in FFPE tissues including all marker genes for glioma but missing important CNVs (*1p/19q*-codeletion and *chr +7/-10*) and p-MGMT methylation (OncoDEEP - Comprehensive biomarker test, 2023) (Table 2). Onco panel, offered by Diatech Pharmacogenetics (Jesi, Italy), has CE-IVD certification and covers 56 genes including usual glioma marker genes such as *BRAF, CDKN2A, EGFR, IDH1, IDH2, NOTCH,* and *TP53* (Marchetti et al., 2021; Eurofins Genomics, 2023). The panel includes several glioma-related genes and misses only the *p-TERT* mutation (Arildsen et al., 2019). CeGaT (CancerPrecision, 2023), missing only *p-TERT* and *H3-3A* for full glioma marker gene analysis; additionally, the possibility of 1p/19q-codeletion detection is not clear. NIMGenetics (Madrid, Spain) offers service for NGS cancer diagnostics, ONCONIM^®^ Biomarker Broad Spectrum, for detecting SNPs, fusions, and SNVs in 52 genes from isolated patient sample DNA or FFPE tissues including *IDH1, IDH2, BRAF* and *EGFR* (Cáncer Somático, 2023). SOPHiA Solid Tumor Solution from SOPHiA GENETICS (Lausanne, Switzerland) has a CE certification and can detect glioma molecular markers such as *IDH1, IDH2, CDKN2A, BRAF, EGFR, TP53, p-TERT,* and *H3-3A* (SOPHiA Solid Tumor Solutions, 2023).

Glioma-specific NGS panels have been developed, however, we were not able to find some of them in the RUO or clinic markets. In this regard, we can merely summarize that there is ongoing research towards a panel specific for patients with glioma and that the market could be soon presented with a new targeted NGS kit. For instance, GlioSeq was distributed by the University of Pittsburgh Medical Center (Pittsburgh, United States) for sequencing 43 genes related to glioma (Nikiforova et al., 2016), and it can probably identify *1p/19q*-codeletion (Roy et al., 2019), although the methodology for identification was not described. CD Genomics (New York, United States) offers RUO Glioma Gene panel for sequencing of 12 glioma marker genes (Table 3) (Glioma Gene Panel Sequencing - CD Genomics, 2023); although it misses many important genes, it has the advantage of low-cost Illumina MiSeq utility. The Glio-DNA panel targets 65 genes including *p-MGMT* methylation, and *p-TERT* mutations, and can possibly identify the important CNVs (*1p/19q*-codeletion and *chr +7/-10*) (Tirrò et al., 2022). Italian researchers designed a glioma panel spanning 13 genes and a *p-TERT* region with the possibility of detecting *1p/19q-*codeletion (Guarnaccia et al., 2022). There is an Illumina MiniSeq compatible comprehensive glioma panel investigating 57 genes for SNPs and indels together with loss of heterozygosity in Chr1p, 6, 7, 9q, 10, 17, and 19q (Lorenz et al., 2020) (Table 3). A 20-gene NGS panel for gliomas compatible with the Ion Torrent platform was developed for molecular profiling of FFPE patient tissues (Zacher et al., 2017). GliomaSCAN is a big NGS panel comprising 232 genes for detecting most of the glioma markers, including SNPs, CNVs (*1p/19q*-codeletion and *chr +7/-10*), and amplifications (*EGFR, PDGFRA*) (Sa et al., 2019; Shin et al., 2020). CliomaSCAN is available on the Chinese market, but it is relatively big to be glioma-specific and ensure cost-effective screening or repeated monitoring and, probably, demands extensive sequencing power to override noise and artificial substitutions.

**TABLE 3:** Glioma-specific NGS panels.

Genes important for glioma molecular profiling	GlioSeq	Glioma gene panel	Glio-DNA	13-Gene glioma panel	57-Gene comprehensive glioma panel	20-Gene glioma-tailored panel	GliomaSCAN
IDH1/2, BRAF, H3-3A (H3F3A), EGFR, TP53, CDKN2A, ATRX. PTEN	+	+	+	+	+	+	+
p-TERT	-	-	+	+	+	+	+
HIST1H3B	+	-	-	+	+	-	+
KIT	+	-	+	-	+	-	+
FUBP1, CDKN2B	+	-	+	-	+	+	+
CIC	+	+	+	-	+	+	+
NOTCH1	-	-	+	-	+	-	+
1p/19q-codel	+	-	+	+	+	-	+
+7chr/-10chr	-	-	+	-	+	-	+
p-MGMT methylation	-	-	+	-	-	-	-
Platform	Ion Torrent PGM	Illumina MiSeq	Ion Torrent PGM	Ion Torrent S5	Illumina MiniSeq	Ion Torrent PGM	Illumina HiSeq 2000
Biological material	brain biopsies, FFPE tissues	genomic DNA, FFPE tissues	FFPE tissues	FFPE tissues	FFPE tissues	frozen tissues, FFPE tissues	snap-frozen tissues
Availability on marker	+	+	-	-	-	-	+

## 5 The ideal test system for the diagnosis of glioma

Preventive screening for glioma is not advisable due to the low incidence and low sensitivity of detecting ctDNA in low grades. However, the molecular phenotyping of gliomas can predict prognosis and temozolomide sensitivity and is also important for disease progression monitoring during relapses.

The ideal test system must 1) provide accurate diagnostics, 2) detect DNA sequence alterations in small amounts of different biological materials, 3) be cost-effective, 4) provide an opportunity to make a diagnosis with the available equipment, and 5) have high positive predictive value (PPV).

### 5.1 Provide accurate diagnosis

**FIGURE 3:**
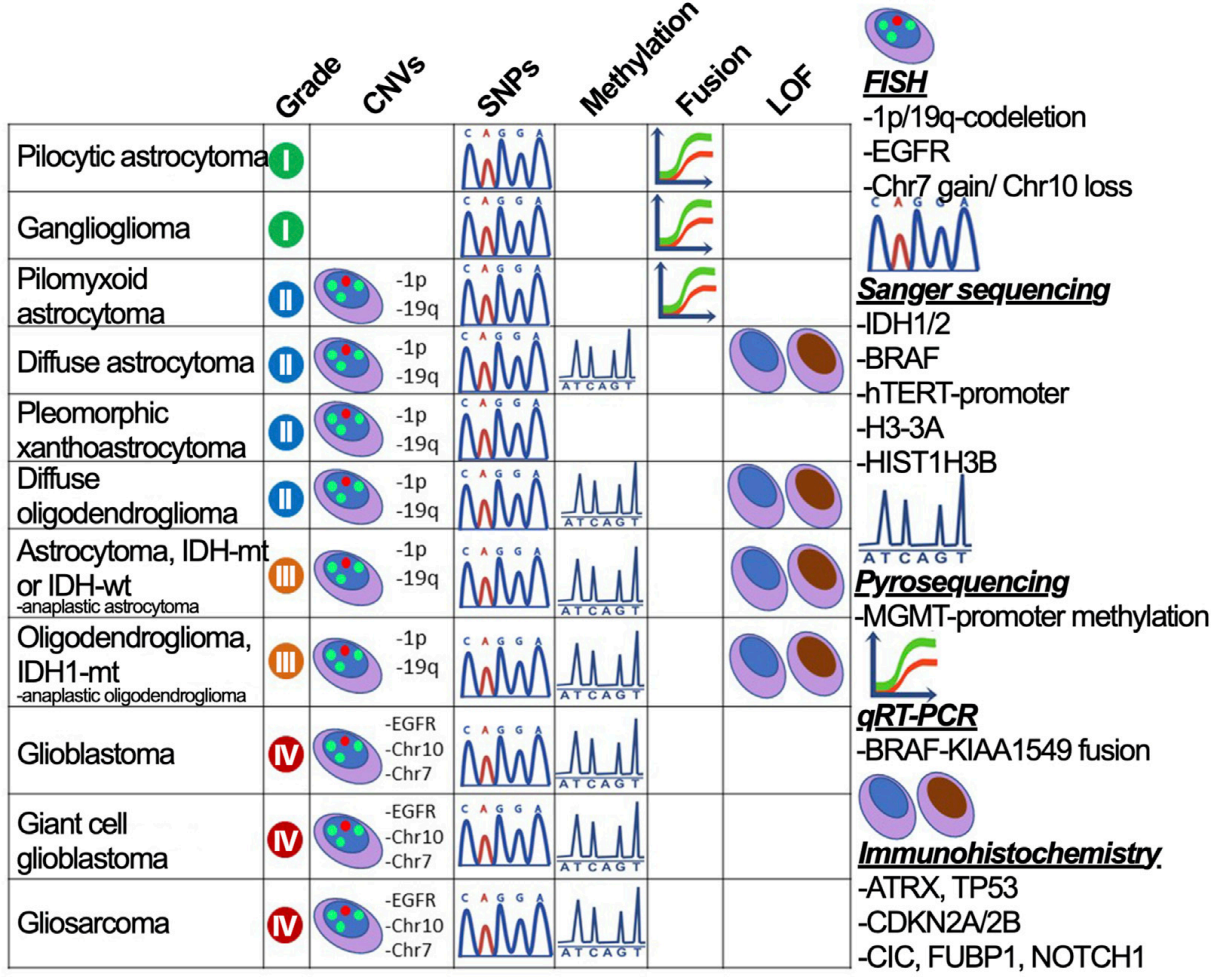
The strategy to divide gliomas into subtypes using gold standard diagnostic procedures. Glioma subdivisions are performed by searching for marker mutations (CNVs, SNPs, fusions, and protein loss-of-function) using methods graphically annotated and described on the right-hand side.

To provide accurate criteria for the diagnosis, the test system needs to incorporate all necessary markers that are sufficient for dividing glioma subtypes. The system should span all glioma marker mutations—DNA sequence alterations—*IDH1* R132, *IDH2* R172, *TP53* (W146, R175, R248, and R273), *p-TERT* (C250T and C228T), *BRAF* V600E, and *H3-3A* K27M; CNAs — *1p/19q*-codeletion and *chr +7/-10*; change in particular gene expression—gain of *EGFR*, *KIT*, and *PDGFRA*, loss of *ATRX*, *PTEN*, and *CDKN2A/B*; small indels—*EGFR*vIII; fusions—*BRAF-KIAA1549* (Figure 3) (Faulkner et al., 2015). However, in case of missing high throughput diagnostic methods, the preliminary assumption stems from the histological subtype and the glioma-specific genes could be enquired sequentially. The strategy for the diagnosis of glioma is described in Supplementary Table S1.

Inherited DNA sequence alterations might predict glioma susceptibility, as a nearly 2-fold increase is seen in relatives of patients with glioma (Hemminki et al., 2009). Several inherited genetic alterations lead to a higher risk of glioma development, for instance, *TP53, TERT, CCDC26,* and *CDKN2B*.

Accurate diagnosis is also related to the specificity and sensitivity of the test system. Modern test systems pursue the highest possible sensitivity to diagnose as many people with the disease as feasible. However, high sensitivity should be balanced by high specificity in picking healthy people (Swift et al., 2020). Nowadays, there is a rising concern about false diagnosis/care/treatment referred to as overmedicalization, which can be avoided by focusing on the test system specificity and patient management in accordance with the real disease symptoms. When creating a test system, one needs to understand not only the purpose of use but also the possibility of misuse (inadvertently) of the diagnostic system, which may lead to unnecessary stress in false-positive patients. A good example is Prostate Specific Antigen, which has a sensitivity of 86% but a specificity of 33% (Swift et al., 2020). It is very good for revealing patients with prostate cancer but 67% of men receive false-positive results. With glioma one needs to be aware that patients diagnosed with glioblastoma tend to reject any treatment due to prominent adverse effects and fast ultimate mortality. Therefore, a diagnostics system should provide the same level of sensitivity and specificity.

### 5.2 Detect DNA sequence alterations in small amounts of different biological material

The gold standard of biological material for the detection of glioma DNA sequence alterations is a tumor piece or biopsy. However, the tumor biopsy in the case of patients with glioma is highly invasive and could cause the deterioration of the disease course. Typically, the glioma diagnosis is performed after the operation by histology and molecular testing, although prior knowledge could be obtained by the MRI visualization of tumor size and location. Nevertheless, the glioma molecular profile before the operation in cases of operation impossibility, or during multiple relapses, could provide additional information on patient prognosis and possible sensitivity to TMZ.

It is possible to use cerebrospinal fluid (CSF) for glioma genotyping as the content of ctDNA from dead glioma cells is sufficient and correlates with the source tumor profile (Miller et al., 2019). This allows for the monitoring of the glioma molecular progression with the relapses without obtaining biopsy samples (Martínez-Ricarte et al., 2018). Histone 3 DNA sequence alterations specific for pediatric gliomas can be identified in CSF using the ddPCR approach with a sensitivity of 87% (Panditharatna et al., 2018).

Another possibility is to use blood for detecting ctDNA, however, the sensitivity of using patient plasma is significantly lower than that of CSF (Juratli et al., 2018). The patients with glioma would have detectable plasma ctDNA only in 20%–50% of cases (Gao et al., 2022). Although, the study conducted in children has shown almost the same sensitivity in detecting H3 histone DNA sequence variants in plasma specimens (90%) compared to CSF (87%) (Panditharatna et al., 2018). Moreover, Guardant360 CDx markets the kit for the molecular characterization of any advanced solid tumor in the patient’s blood plasma (GUARDANT368 cDX, 2023), but it misses the genes for oligodendroglioma diagnosis (Table 2).

Companies provide devices; for instance, chemagic™ instruments offered by PerkinElmer (cfDNA Isolation and Analysis Instruments|PerkinElmer, 2023), Roche sells hybridization-based enrichment kits, and QIAgen features a technique based on single primer extension (Lam et al., 2020).

There is an emerging technique called “sonobiopsy” to facilitate the ctDNA release in the blood with the means of sonication disruption of tumor cells and blood-CSF barrier, which was shown to increase the sensitivity of the glioma marker DNA sequence alteration detection for *EGFR*vIII and *p-TERT* SNVs (Pacia et al., 2022).

### 5.3 Be cost-effective (availability of a non-inexpensive test)

NGS technology is the most effective in terms of profiling patient materials and revealing SNVs as well as small indels. However, there is still no standard protocol to detect large chromosomal aberrations, such as *1p/19q*-codeletion and *chr +7/-10,* indispensable for glioma diagnosis. Despite a decreasing trend in NGS costs during the last few years, the costs of big pan-cancer panels are not affordable for routine patient surveillance, and big panel sizes could affect the sensitivity of marker identification and decrease the signal-to-noise ratio (Cohen et al., 2018). Thus, the usage of FDA-certified and CE-IVD-certified multicancer panels (Table 2) for glioma profiling/monitoring/prediction is questionable.

Additionally, there is no possibility to establish a sequencing unit and provide a linked technician and bioinformatician at every hospital. Nowadays, NGS can substitute all single-gene evaluation approaches. For instance, the diagnosis of glioma is based on inquiring about DNA sequence alterations in *IDH, TP53,* and *EGFR* and the presence of *1p/19q*-codeletion. For this purpose, we can use Sanger sequencing, qPCR, immunohistochemistry for SNPs and in FISH for chromosomal aberrations, however, NGS can replace all the mentioned techniques and reveal additional non-canonical polymorphisms.

### 5.4 Provide an opportunity to make a diagnosis with the available equipment

There is a possibility to use so-called targeted NGS focused on precise gene regions, for example, the cancer hot-spot panel traded by Ion Torrent covering 50 oncogenes with approximately 2,800 COSMIC mutations (Ion AmpliSeq™ Cancer Hotspot Panel v2, 2023), including glioma related DNA sequence alterations (Table 2). The targeted NGS technology allows for an increase in the number of sequenced patients and could lower the number of spent reagents per run (Plasmodium Diversity Network Africa et al., 2019).

NGS market in percentage of revenue for 2020 is separated as follows: North America — 40.5%, Europe — 28.3%, Asia-Pacific — 18.9%, South America — 7.8%, and Middle East and Africa — 4.5%. Some institutions in developing countries outsource the NGS to specialized laboratories in more developed countries (Ghilamicael et al., 2018; Tawfik et al., 2018; Mulenga et al., 2020). With this in mind, NGS technology is not available for patients in most regions partially due to difficulties with expensive equipment.

Nevertheless, the qPCR market is spread in major regions of the world, including North and Latin America, Europe, South and East Asia, Oceania, Middle and East, and Africa. The major advantage is that qPCR technology does not require high-quality instruments, special large rooms in labs, as FISH does, or a microscope with expensive antibodies, as IHC does.

### 5.5 Have a high positive predictive value (PPV)

CNS tumors are the most common cancer in the pediatric population (age range of 0–14 years) (Ostrom et al., 2019; Hauser, 2021). However, glioma is a rare type of cancer in the general adult population, and the probability of its occurrence peaks at the age of 36–59 years for different types (Molinaro et al., 2019). Hence, it is important that the healthcare system is prepared to screen only relevant subgroups of populations. These measures would prevent overdiagnosis and overtreatment.

## 6 Diagnostic strategy for the “ideal test system”

Molecular diagnostics requires a developed infrastructure. There is a need for stable electricity, low-temperature storage of mixes and samples, nuclease-free water, and trained personnel with relevant working time and wages. This is a problem that is still faced by developing countries and even developed countries in areas with low infrastructure (Abou Tayoun et al., 2014).

A survey conducted in 314 centers in 48 countries on the use of molecular diagnostics for gliomas has shown that molecular diagnostics is used in 235 centers (74.8%), and participants from all centers in 12 out of 48 countries (25%) stated that they do not have access to methods of molecular diagnosis of brain tumors. These 75% of centers that use molecular diagnostics in their clinical practice widely use such methods as FISH and/or CISH (216 centers, 69%), whereas 194 centers out of the 75% can perform other molecular diagnostics methods besides CISH/FISH aimed at analyzing the following: status *1p/19q* (72% of all centers), the methylation status of the *MGMT* promoter (53%), *BRAF* sequencing (50%), and *IDH1* mutation (47%) (Andreiuolo et al., 2016).

### 6.1 The pitfalls to performing high throughput diagnostics

The estimated cost of establishing an NGS facility ranges between $100K to $700K U.S. dollars (El-Metwally et al., 2014). On average, to cover the country’s needs for sequencing there should be one sequencer per 2.4 million inhabitants. This is complicated to reach, especially for countries with large populations or countries with large skews in the distribution of the population over the territory.

The salary for NGS unit specialists in Italy is €61,670/year, €61,601/year, and €73,047/year for laboratory technicians, biologists, and pathologists, respectively (Pruneri et al., 2021). In Spain, a specialist technician is paid €33,155/year and a physician-oncologist receives €94,952/year (de Alava et al., 2022). The average salary of a molecular biologist in India is €3,748/year and for a bioinformatician, it is €7,369/year (Next-Generation Sequencing NGS Salary in India | PayScale, 2023). The gross salary in NGS facilities in lower and middle-income countries varies significantly, for instance, in South America, the average wage ranges from US$31,800 to US$45,500/year, while in China it ranges from US$39,000 to US$48,000/year, in Brazil it ranges from US$20,900 to US$37,000/year, in Russia it ranges from US$18,000 to US$31,800/year, and in India it ranges from US$11,700 to US$20,700/year. The information is provided by the website SalaryExpert (Institute, 2023).

Furthermore, establishing an NGS facility in a developing country could be far more expensive because of the added value of shipments, customs, and profit margin for local companies (Helmy et al., 2016). Typically, due to logistic issues, there are not many possibilities of delivering reagents that have to be transported in low-temperature conditions. Additional challenges include the lack of instrument maintenance personnel, the inability to quickly repair broken devices, the absence of qualified staff (especially for small towns), and limited access to up-to-date scientific literature (Helmy et al., 2016). The solution could be to establish/improve the research supply logistics, remove import duties on products for cutting-edge scientific research (NGS, mass spectrometry, and plasmids for gene editing), or elaborate diagnosis with other genotyping procedures, such as qPCR and allele-specific PCR).

### 6.2 The availability of NGS services throughout the world

**FIGURE 4:**
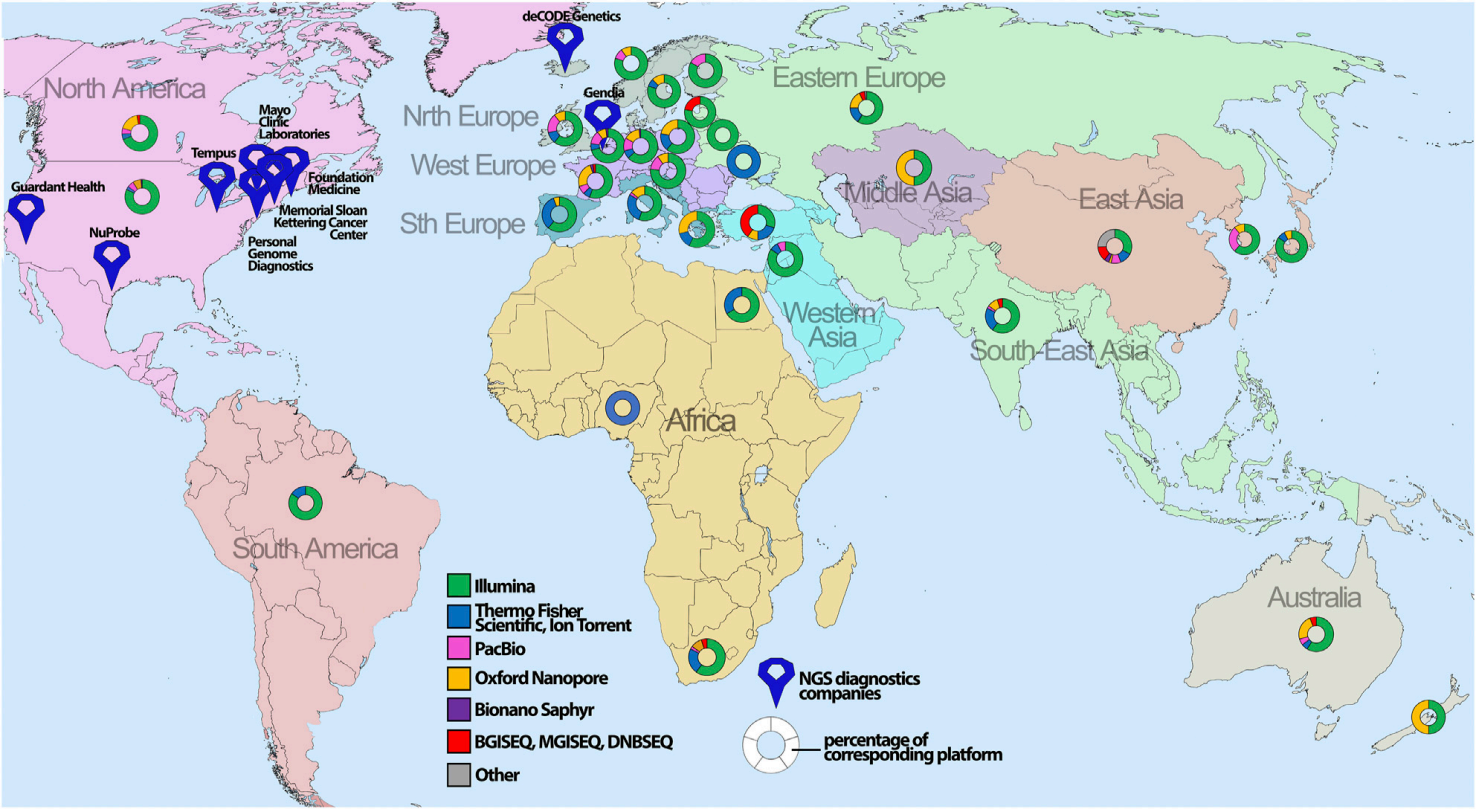
The global geographical distribution of sequencing platforms and technologies. The percentage of the corresponding sequencing platform analyzed for certain countries is depicted with map symbols of different colors and shapes showing the geographical location. The color annotates different NGS platforms, whereas size indicates the number of sequencers for the corresponding platform.

In general, sequencing availability extremely varies throughout the world, with the highest concentration in the United States and Western Europe (Figure 4). Specifically, Illumina is the platform of choice for hospitals globally (Supplementary Figure S3) with the most frequent Illumina MiSeq accessibility due to relative low-cost of routine handling and high usage for clinical diagnostics. The second most popular platform is Oxford Nanopore Technologies with the device of choice, MinION, being utilized for long-read sequencing and usually employed by scientific institutions/universities for *de novo* genome assembly or low-cost WGS.

African countries face difficulties in providing patients with NGS services because of the pitfalls mentioned above. Nevertheless, the sequencing of drug-resistant tuberculosis strains was performed utilizing Illumina iSeq 100 at Redeemer’s University, in Ede, Nigeria. (Olawoye et al., 2021). The sequencing capacity of North Africa is concentrated in Egypt; for instance, Cairo University (Cairo, Egypt) possesses Illumina MiSeqDx and Ion Proton System, while Suez Canal University (Ismailia, Egypt) exploits Illumina MiSeq. Compared to the rest of Africa, South Africa has a much larger NGS capacity, and private companies utilize NGS for screening procedures and present a broad variety of NGS platforms from Chinese MGI-SEQ2000, all kinds of Illumina platforms to WGS sequencers such asPacBio Sequel II (Figure 4; Supplementary Figure S2).

Although India is considered a lower middle-income country, it possesses many NGS facilities (Supplementary Table S2). However, there is an issue with providing complete information about the advertised clinical services, thus some companies do not provide a description of the system they use for performing NGS analysis and do not mention whether they outsource this task to third parties (Salwan et al., 2020; Wen et al., 2020; Dash et al., 2021; Poonia et al., 2023). Indian Council of Medical Research claims that 84 institutes throughout the country possess an NGS facility (Next Generation Sequencing Capacity of India, 2023), however, it was difficult to confirm this as research institutions do not typically advertise the actual availability of their sequencing capacity. According to the available scientific research papers, several institutions were verified to have working NGS units (Supplementary Table S2). On the other hand, India has a wide variety of relatively small companies featuring NGS services. For instance, Genotypic Technology Pvt. Ltd. markets NGS employing the Nanopore platform (Next Generation Sequencing—Genotypic Technology Pvt. Ltd, 2023). Igenomix India offers whole genome sequencing technology for 24,000 genes but does not describe their platform. Eurofins Genomics India provides an opportunity to sequence a genome using Illumina NextSeq 500, Illumina HiSeq 2500 and Illumina NovaSeq. MedGenome Labs (Bengaluru, India) has a larger offer for various sequencing options, offering Illumina HiSeq X Ten, Illumina HiSeq 4000, Illumina NextSeq 500, Illumina MiSeq, Illumina HiSeq 2500, Illumina iScan, and Ion Proton System. Xcelris Labs (Ahmedabad, India) offers NGS sequencing on Illumina NextSeq500, Illumina MiSeq, GS FLX Titanium by Roche, Ion Proton System, and Roche 454 GSFLX + for pyrosequencing. Nucleome Informatics (Hyderabad, India) powers the genome sequencing on Bionano Saphyr, PacBio Sequel II, Illumina NovaSeq/HiSeq/iScan, as well as targeted gene sequencing with implication of Illumina MiSeq and Thermo Fisher GeneTitan. Biokart India provides transcriptome and genome sequencing, but does not describe properly the NGS platform used for these services. According to available publications, they have in use Illumina Miseq (Sajid et al., 2021) (Figure 4; Supplementary Table S2).

The Chinese NGS market is filled by big companies with targeted panels for prenatal diagnostics, cancer diagnostics, and diagnosis of rare hereditary diseases. For example, Berry Genomics markets an analog for Illumina NovaSeq500: NextSeq CN500, along with Illumina, PacBio, and Bionano devices (251). Similarly, Annoroad Gene Technology (Beijing) developed a sequencing machine analogous to Illumina NextSeq 500: NextSeq 550AR. CapitalBio Technology (Beijing) and Daan Gene (Guangzhou) market analogous devices to Ion Torrent S5: BioelectronSeq 4000 Gene Sequencer and DA8600, respectively (Zeng et al., 2022). Genetron Health (Beijing) provides an option for the Ion Torrent platform featuring their sequencers GENETRON S5 and GENETRON S2000 along with Genetron Chef System. BGI Group offers MGI DNBseq™ technology along with BGISEQ-500 and more recent MGISEQ-2000 devices for transcriptome analysis, with the latter showing a comparable performance with the Illumina NextSeq 500 (Zeng et al., 2022), as well as DNBSEQ-T7 and DNBSEQ-G400 for WGS (Kanzi et al., 2020). GeneMind Biosciences (Shenzhen) developed sequencers: GenoLab M based on reversible termination of the fluorescent sequencing to identify optical signals from bases and Genocare 1600, which has the same optical signal technology but is implemented for single molecule sequencing. Shenzhen HYK Gene Technology features two sequencing devices: PSTAR-IIA and SeqExpert III-A without the description of the sequencing technology. Anxuyuan Biotechnology (Axbio Inc.) offers a fourth-generation sequencer AXP100 by combining Nanopore technology with single-molecule sequencing on the Biosensor-CMOS Platform implementing capacity sensor for proteins (Alhoshany et al., 2017). GenePlus-Suzhou (Suzhou) sells Gene + Seq-2000 and Gene + Seq-200 gene sequencers with the integrated DNBSEQ core technology (Wu et al., 2022). TIANGEN Biotech (Beijing) features transcriptome and genome sequencing using Illumina or BGI platforms. Biomarker Technology Corporation has a subdivision, BNKGene (Beijing), offering a wide variety of sequencing services, such as whole-transcriptome sequencing (WGS/WTS) on all kinds of platforms, e.g., PacBio Sequel, Nanopore PromethION, and GridION X5 MinION, Illumina NovaSeq, BGI DNBSEQ, and low-cost Bionano Irys. Guangzhou Huayin Health Medical Group (Guangzhou) has a subdivision, Huayin Biology, that trades sequencing services on Illumina HiSeq 3000, Illumina NextSeq 500, Illumina MiSeq, IonProton, and IonPGM. Novogene (Beijing) provides whole- and targeted genome sequencing on a variety of platforms, such as Illumina NovaSeq 6000, HiSeq X Ten and HiSeq 4000, PacBioSEQUEL II/IIE, and Nanopore PromethION. 3D Medicines (Shanghai) offers Illumina NovaSeq 6000 for sequencing services (Figure 4; Supplementary Table S2).

Similarly, the Russian NGS market is comprised of companies providing NGS devices or services and research institutions featuring services for exome/genome sequencing and data analysis. Russian companies do not provide the proprietary developed technology for sequencing but propose all kinds of platforms for WES/WGS such as Illumina, Ion Torrent, Nanopore, and DNBSEQ featured for research by R-pharm and Helicon, for clinical use by Genotek and Genoanalytica (Supplementary Table S2).

The global market for Europe and the United States is well characterized, thus we decided to point out the overall sequencing market research. Normanno et al. estimated the availability of oncological biomarker investigation in Europe. Multibiomarker test access through NGS services is high in Austria, Belgium, Cyprus, Denmark, Finland, France, Germany, Ireland, Portugal, Sweden, and the United Kingdom (100% of multi-biomarker access). Medium availability of NGS services was observed in Croatia, Czechia, Hungary, Netherlands, and Spain (75%–100% of multi-biomarker access), whereas there was low availability, accounting for less than 75% of multi-biomarker access, in Bulgaria, Estonia, Greece, Italy, Latvia, Lithuania, Luxembourg, Poland, Romania, Slovakia, and Slovenia (Normanno et al., 2022). In terms of support, governmental funding is received in a few European countries, namely, the United Kingdom, Belgium, Denmark, and the Netherlands (Phillips et al., 2021). Australia also possesses a governmental program for NGS development. Considering North America, WGS is not reimbursed while WES is funded in several provinces of Canada. However, the utility of WES is questionable as the time to receive results is delayed (2–6 months) and Canada outsources most of WES analysis abroad as the proper clinical NGS infrastructure is missing. The United States has private/public insurance coverage for clinical WGS and WES, which has been successfully used by more than half of insured individuals (Phillips et al., 2021).

Nevertheless, the largest NGS market is in the United States. Major companies such as Illumina, Thermo Fisher Scientific, Myriad Genetics, Roche, Bio-Rad Laboratories, and Perkin Elmer provide NGS services for both clinic and research. Alternative NGS operators are Oxford Nanopore Technologies in the United Kingdom, QiaGen from Germany, and Macrogen from South Korea. China’s NGS market stands separately, with major companies such as MGI/BGI, Burning Rock, Novogene, and Berry Genomics, although Chinese offers of NGS services often provide an incomplete description of the sequencing technology or panels.

## 7 Discussion

In this review, it was observed that there is no comprehensive kit or test system to profile all the variety of glioma marker DNA sequence alterations, including SNVs, indels, amplifications, fusions, CNVs, and methylation status. First, there is no platform fully covering the complexity of glioma genetic variants because there is no methodology encompassing all the possible mutations in one single experiment. NGS panels are now highly invested in incorporating the most clinically relevant glioma mutations. However, so far, most of the targeted glioma panels reported in scientific papers remain inaccessible for even the RUO biomedical market, not to mention for *in vitro* clinical diagnosis.

Glioma molecular diagnostics provides an opportunity for precision oncology but remains an issue. To profile all subtyping DNA sequence alterations in a clinical lab there is a need to establish separate difficult procedures for SNPs (Sanger sequencing or qPCR), CNVs (FISH), amplification, or loss-of-function (IHC and *MGMT*-promoter methylation—pyrosequencing). This difficulty could be partially circumvented by building a clinical NGS testing facility and usage of pan-cancer panels certified for diagnosis. However, this approach is still expensive, because of the cost of building an appropriate unit and low demand for screening the general population due to low incidence in people under 60 years old (peaking incidence rate age is 70–79 years for glioblastoma and astrocytoma and 30–49 years for oligodendrogliomas) (Wang G.-M. et al., 2022). Additionally, there is no clinically approved glioma-specific NGS panel, and none of the developed panels could identify *MGMT*-promoter methylation relevant for predicting temozolomide responsiveness.

For prognostic and treatment response prediction, physicians could implement the shortened list of molecular markers, mainly DNA sequence alterations in *IDH1/2* affecting temozolomide response, *EGFR* amplification or truncation affecting the use of anti-*EGFR* therapy in glioma, and *p-TERT* mutations conferring a poor glioma prognosis (Killela et al., 2014). Both qPCR and ddPCR could be employed for SNP revealing, with the latter being more sensitive for determining mutations in blood and CSF ctDNA (Olympios et al., 2021). During the SARS-CoV-2 pandemic, the availability of ddPCR and qPCR has grown profoundly, spreading to small hospitals. The ddPCR segment is growing even faster, although ddPCR’s high cost still precludes dissemination. However, there is no FDA-approved qPCR kit for detecting SNPs in patients with glioma, as the Abbott RealTime *IDH1/2* kits are approved for patients with acute myeloid leukemia. The European market has clinically certified qPCR kits for *IDH1/2* mutation detection from Qiagen and EntroGen, and *MGMT*-promoter methylation kits from EntroGen and Genmark Sağlık Ürünleri. Genetron Health provides qPCR kits for identifying DNA sequence alterations in *IDH1* and *p-TERT* approved by Chinese NMPA. Thus, glioma molecular profiling in clinics is either performed by diverse procedures (sending to clinically approved diagnostic labs providing services for mutation revealing) or represents an unmet need.

We suggest that the market should not consolidate hope only in the NGS segment but rather consider other perspectives for clinical development, such as qPCR, ddPCR, and MassArray, which could be developed for liquid biopsy and incorporate a wide spectrum of glioma-related mutations simultaneously.
