# Supplementary material for: Comprehensive clinical assays for molecular diagnostics of gliomas: the current state and future prospects
Source: Front Mol Biosci. 2023 Oct 16;10:1216102. doi: 10.3389/fmolb.2023.1216102 (PMC10613994; doi:10.3389/fmolb.2023.1216102)
Supplement: Supplementary file 1 [file DataSheet1.docx]

Supplementary Material

All tumors were divided into six different families, four of these families describe different types of gliomas. Adult-type diffuse gliomas now include 3 types of tumors: Astrocytoma, IDH-mutant; Oligodendroglioma, IDH-mutant and 1p/19q-codeleted; Glioblastoma, IDH-wildtype. Pediatric-type diffuse gliomas now divided into low-grade and high-grade types. Pediatric-type low-grade diffuse gliomas are expected to have good prognosis and now include Diffuse astrocytoma, MYB- or MYBL1-altered; Angiocentric glioma; Polymorphous low-grade neuroepithelial tumor of the young and newly recognized Diffuse low-grade glioma, MAPK pathway-altered. Pediatric-type diffuse high-grade gliomas are expected to have aggressive behavior. This family comprise Diffuse midline glioma, H3 K27-altered; Diffuse hemispheric glioma, H3 G34-mutant; Diffuse pediatric-type high-grade glioma, H3-wildtype and IDH-wildtype; Infant-type hemispheric glioma. Circumscribed astrocytic gliomas were named “circumscribed” as opposed to the “diffuse'' tumors in the first three groups. This family consist of Pilocytic astrocytoma; High-grade astrocytoma with piloid features; Pleomorphic xanthoastrocytoma; Subependymal giant cell astrocytoma; Chordoid glioma and Astroblastoma, MN1-altered, which has been specified as “MN1-altered” to draw more attention to MN1 mutation, a transcriptional coregulator involved in the development of acute myeloid leukemia and meningioma, which in turn is now used to define the lesion (https://doi.org/10.1093/neuonc/noab106).


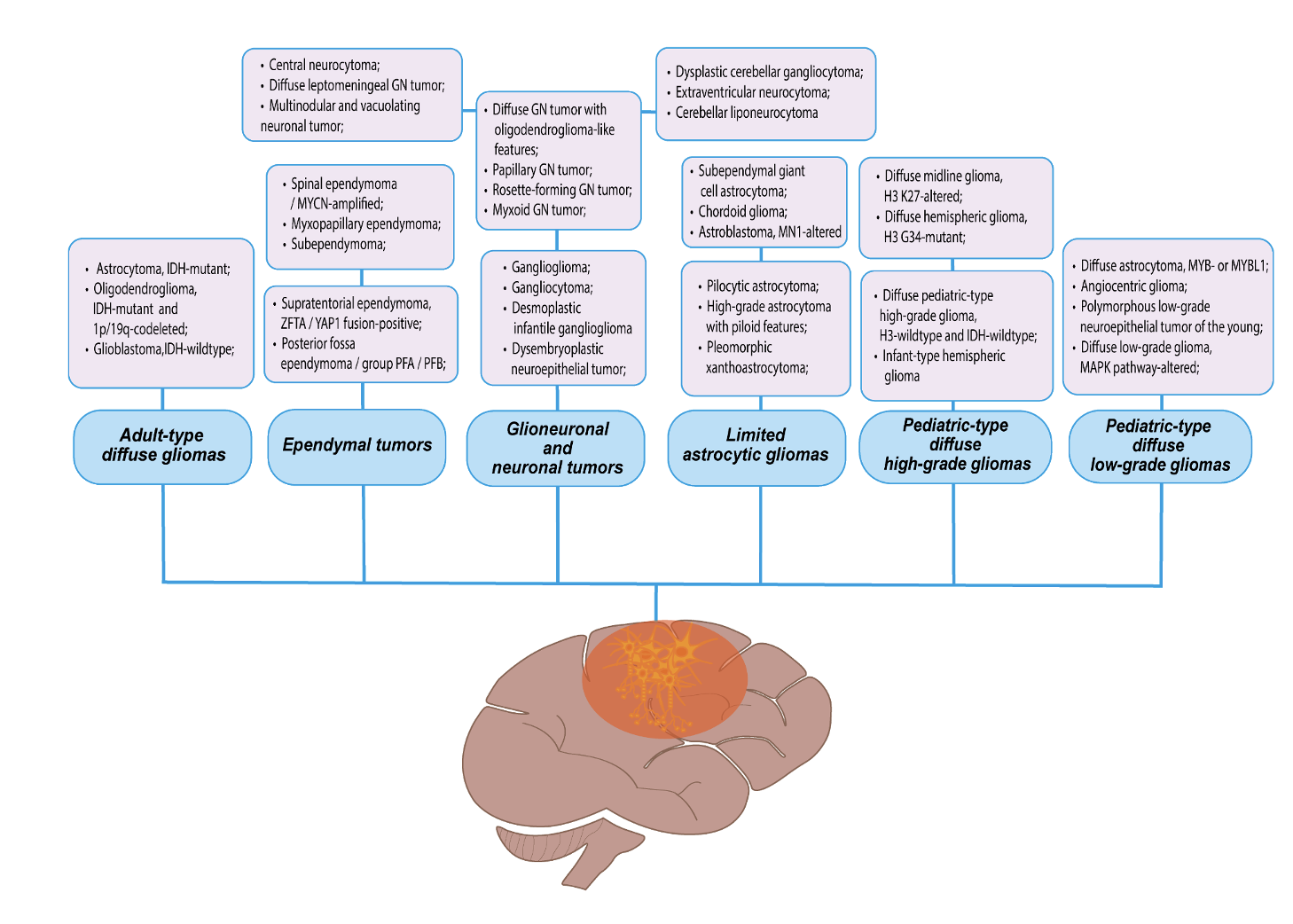


**Supplementary Figure S1.** Brain tumor classification.


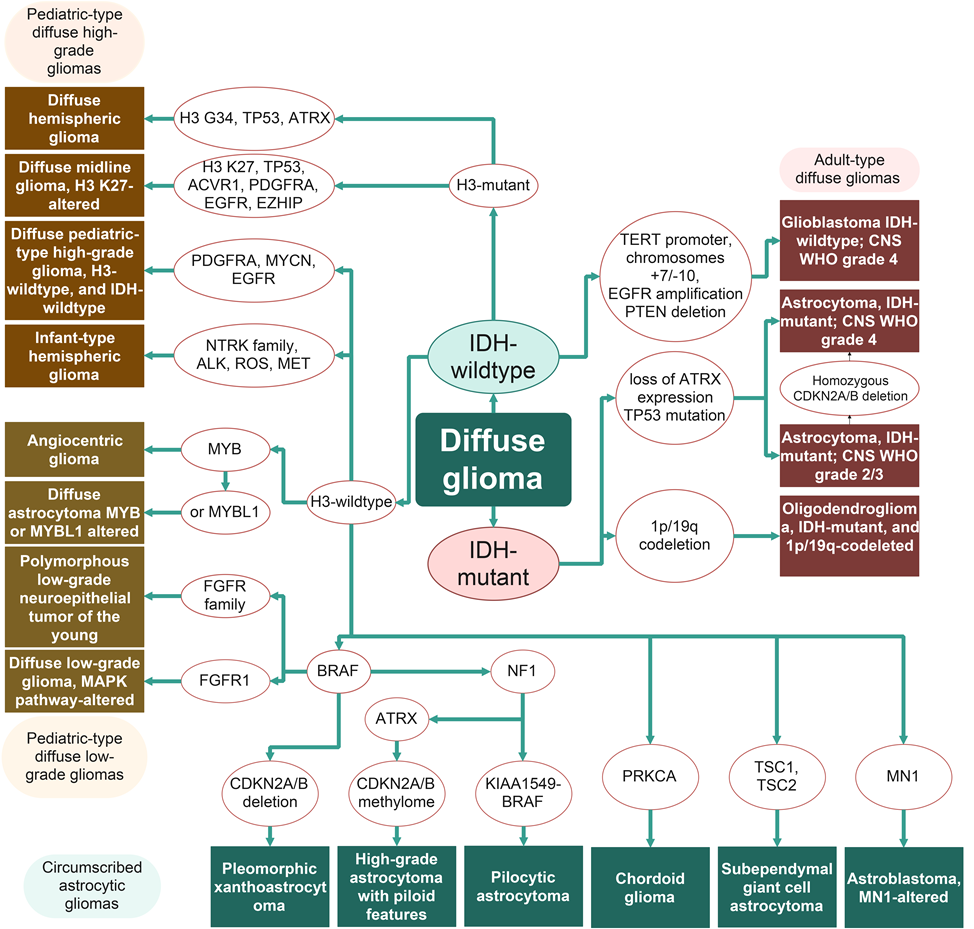


**Supplementary Figure S2.** The schematic representation of the glioma diagnosis in accordance with the mutational profile. The mutated genes were depicted in circles, the corresponding diagnosis presented in squares with different colors for separate glioma groups.


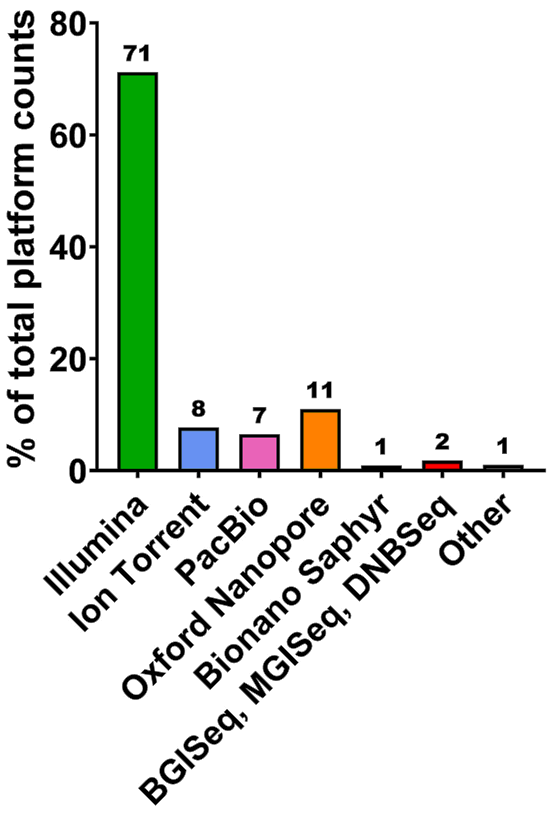


**Supplementary Figure S3.** Worldwide distribution of sequencers by platform. The data depicted as a percentage of all devices of six main “brands” (Illumina, Ion Torrent, Pacific Bioscience – PacBio, Oxford Nanopore Technologies, BGISeq/MGISeq/DNBSeq) found on the webpages of Universities, Scientific Institutions, Hospitals. Refers to Supplementary Table S2.

**Supplementary Table S1.** The strategy for glioma molecular diagnostic.

| Grade | Type | 1p/19q-codeletion | SNP in IDH1/ IDH2/ BRAF/ hTERT-promoter/ H3-3A /HIST1H3B | MGMT methylation | BRAF –KIAA1549 fusion | EGFR amplification/variant III; 10 chromosome loss/ 7 chromosome gain, KIT gain |
| --- | --- | --- | --- | --- | --- | --- |
| I | Pilocytic astrocytoma |  | V |  | V |  |
| I | Ganglioglioma |  | V |  | V |  |
| II | Pilomyxoid astrocytoma | V | V |  | V |  |
| II | Diffuse astrocytoma | V | V | V |  |  |
| II | Pleomorphic xanthoastrocytoma | V | V |  |  |  |
| II | Oligodendroglioma | V | V | V |  |  |
| III | Astrocytoma, IDH-mt or IDH-wt | V | V | V |  | V |
| III | Olidogendtoglioma, IDH1-mt | V | V | V |  |  |
| IV | Glioblastoma |  | V | V |  | V |
| IV | Giant Cell glioblastoma |  | V | V |  | V |
| IV | Gliosarcoma |  | V | V |  | V |

**Supplementary Table S2.** The availability of NGS units worldwide by Geography.

| Country | City | Institution/Organization | Platform | Reference |
| --- | --- | --- | --- | --- |
| Nigeria | Ede | Redeemer's University | Illumina iSeq 100 | https://doi.org/10.1038/s41598-021-96956-7 |
| Egypt | Cairo | Cairo University | Illumina MiSeqDx | <https://jenci.springeropen.com/articles/10.1186/s43046-021-00067-3#Sec2>, https://onlinelibrary.wiley.com/doi/10.1002/mgg3.1992 |
|  | Cairo | Cairo University | Ion Proton™ System | https://www.sciencedirect.com/science/article/pii/S2090123220300631 |
|  | Ismailia | Suez Canal University | Illumina MiSeq | https://journals.plos.org/plosone/article/authors?id=10.1371/journal.pone.0265884 |
| South Africa | Cape Town | South African Medical Research Council | MGI-SEQ2000 | https://doi.org/10.1038/s41598-020-79794-x |
|  | Cape Town | University of Cape Town | Illumina HiSeq2500 | https://www.ncbi.nlm.nih.gov/pmc/articles/PMC5132076/ |
|  | Durban | Kwazulu-Natal Research Innovation and Sequencing Platform | Ion Torrent; Illumina 2x MiSeq, 2x NextSeq 500, HiSeq; Oxford Nanopore MinION | http://www.krisp.org.za/toys.php?category=Sequencers |
|  | Pretoria | Agricultural Research Council | Illumina HiSeq2500, 2x MiSeq | https://www.arc.agric.za/Pages/BTP/Next-Generation-Sequencing.aspx |
|  | Pretoria | Inqaba Biotechnical Industries (Pty) Ltd | PacBio Sequel II; Illumina MiSeq, NextSeq | https://inqababiotec.co.za/services/ngs/ |
|  | Pretoria | University of Pretoria | Ion Proton™ System | 10.1017/S0950268814000818, https://www.up.ac.za/faculty-of-natural-agricultural-sciences/news/post_1646310-ion-torrent |
|  | Stellenbosch | Stellenbosch University | Ion Proton™ System | <https://www.mdpi.com/1422-0067/18/2/467/htm>, https://bmcmedgenet.biomedcentral.com/articles/10.1186/s12881-020-0953-1#Sec2 |
| India | Ahmedabad | Xcelris Labs Ltd. | Illumina NextSeq500, MiSeq; GS FLX Titanium by Roche; Ion Proton | http://www.xcelrisgenomics.com/NGS-WholeTranscriptomeSequencing.html |
|  | Bangalore | Eurofins Genomics India Pvt Ltd | Illumina NextSeq 500, HiSeq 2500, NovaSeq | https://www.eurofins.in/genomics/next-generation-sequencing/genome-sequencing/ |
|  | Bangalore | Tata Institute of Fundamental Research, National Center of biological Sciences | Illumina HiSeq 2500, MiSeq | https://www.ncbs.res.in/research-facilities/genomics |
|  | Bangalore | Bionivid Technology Pvt. Ltd. | Illumina HiSeq X Ten | https://link.springer.com/article/10.1007/s00425-021-03625-0 |
|  | Bangalore | MedGenome Labs | Illumina HiSeq X Ten, HiSeq 4000, NextSeq 500, MiSeq, HiSeq 2500, iScan; Ion Proton | https://diagnostics.medgenome.com/research-services/ |
|  | Bangalore | Genotypic Technology Pvt. Ltd. | Oxford Nanopore MinION, GridION, PromethION | https://www.genotypic.co.in/next-generation-sequencing/ |
|  | Bangalore | Biokart India Pvt Ltd | Illumina Miseq | <http://biokart.com/ngs-based-services/>; https://www.ncbi.nlm.nih.gov/pmc/articles/PMC8740325/ |
|  | Bangalore | Centre for Cellular And Molecular Platforms (C-CAMP) | Illumina HiSeq 1000, MiSeq; Ion Proton, Roche 454 GS FLX | https://www.ccamp.res.in/NGS-Genomics |
|  | Bhubaneswar | Indian Council of Medical research (ICMR) | Illumina NextSeq 550 | doi: <https://doi.org/10.1101/2022.04.11.487831> |
|  | Chandigarh | Postgraduate Institute of Medical Education and Research | Ion Proton | <https://www.nature.com/articles/s41598-022-14522-1#Sec2> |
|  | Gurgaon | Medanta-The Medicity | Ion Torrent PGM | <https://www.indianjcancer.com/article.asp?issn=0019-509X;year=2022;volume=59;issue=2;spage=218;epage=222;aulast=Mehra> |
|  | Hyderabad | Centre for Cellular & Molecular Biology | Illumina NovaSeq 6000, Oxford Nanopore PromethION P24, MGI-2000 | <https://www.ccmb.res.in/Facilities-Services/Research-Facilities/Next-Generation-Sequencing> |
|  | Hyderabad | Nucleome Informatics Pvt Ltd | PacBio Sequel II; Thermo Fisher GeneTitan; Illumina NovaSeq 6000, HiSeq 2500, MiSeq, iScan | <https://www.nucleomeinfo.com/technologies-we-work-on/> |
|  | Hyderabad | Ocimum Biosolutions | Illumina MiSeq, HiSeq 2000; Ion Torrent S5; PacBio RS | https://ocimumbio.com/next-gen-sequencing/ |
|  | Kharghar | Advanced Centre for Treatment, Research and Education in Cancer (ACTREC) | Illumina HiSeq 1500, Ion Torrent PGM | <https://actrec.gov.in/cri-research-support-facility-detail/74> |
|  | New Delhi | Guru Gobind Singh Indraprastha University | Illumina HiSeq 4000 | <https://www.sciencedirect.com/science/article/pii/S0888754321004146?via%3Dihub> |
|  | New Delhi | Institute of  Genomic and Integrative Biology (CSIR–IGIB) | Illumina HiSeq2000 | <https://pubmed.ncbi.nlm.nih.gov/33980730/> |
|  | New Delhi | Council of Scientific and Industrial Research (CSIR) | Illumina NovaSeq 6000, MGISeq 2000 | <https://www.ccmb.res.in/Facilities-Services/Research-Facilities/Next-Generation-Sequencing> |
|  | Pilani | Birla Institute of Technology and Science (BITS) | Illumina HiSeq 2000 | <https://link.springer.com/article/10.1007/s12672-021-00441-6#Sec2> |
|  | Puducherry | Jawaharlal Institute of Postgraduate Medical Education and Research (JIPMER) | Illumina HiSeq 2500 | <http://journal.waocp.org/article_90180_3449ccafeac59e792f94b66bb2381ff0.pdf> |
| China | Beijing | Berry Genomics | NextSeq CN500 Gene Sequencer, Illumina NovaSeq 6000, HiSeq 2500, MiSeq; PacBio Sequel/ Sequel II, Bionano Saphyr, Bionano Irys | <https://www.berrygenomics.com/column/88/> |
|  | Beijing | Biomarker Technologies Corporation | Illumina HiSeq XTen | <https://www.frontiersin.org/articles/10.3389/fmicb.2020.602039/full#h3> |
|  | Beijing | Genetron Health | GENETRON S5, GENETRON S2000 | <https://en.genetronhealth.com/products-cancerOrMonitor.html> |
|  | Beijing, Suzhou | GenePlus-Suzhou | Gene+ Seq-2000 | <https://www.geneplus.cn/geneplus/trans/toCoreTechnology?page=IVD_seq2000> |
|  | Beijing | Novogene | Illumina NovaSeq 6000, HiSeq X Ten, and HiSeq 4000; PacBio Sequel II/IIE; Nanopore PromethION | <https://en.novogene.com/technology/platforms/> |
|  | Beijing | Annoroad Gene Technology | NextSeq 550AR | <https://www.annoroad.com/en/products/IR/> |
|  | Beijing | CapitalBio Technology | BioelectronSeq 4000 Gene Sequencer | <https://www.capitalbiotech.com/product/23/%E4%BB%AA%E5%99%A8%E5%B9%B3%E5%8F%B0/%E9%AB%98%E9%80%9A%E9%87%8F%E6%B5%8B%E5%BA%8F%E5%B9%B3%E5%8F%B0.html> |
|  | Chongqing | Children’s Hospital of Chongqing Medical University | Illumina HiSeq 2000 | <https://doi.org/10.5114/aoms/130993> |
|  | Guangzhou, Hong Kong | Guangzhou Huayin Health Medical Group Co., Ltd | Illumina HiSeq 3000, NextSeq 500, MiSeq; IonProton, IonPGM | <http://www.huayinlab.com/ServicesDetail.aspx?c=0306> |
|  | Guangzhou | Daan Gene | DA8600 | 10.12998/wjcc.v10.i15.4761 |
|  | Shanghai | 3D Medicines Inc. | Illumina NovaSeq 6000 | <https://www.frontiersin.org/articles/10.3389/fonc.2022.898916/full> |
|  | Shenzhen | BGI Group; MGI Tech Co. | BGISEQ-500, MGISEQ-2000, DNBSEQ-T7, DNBSEQ-G400, DNBSEQ-G99, DNBSEQ-G50 | <https://www.bgi.com/us/sequencing-services/dna-sequencing/whole-genome-sequencing/>, <https://bmcgenomics.biomedcentral.com/articles/10.1186/s12864-019-5965-x>; <https://en.mgi-tech.com/products/instruments_info/5/> |
|  | Shenzhen | GeneMind Biosciences | Genocare 1600, GenoLab M | <http://www.genemind.com/yiqipt.aspx?nid=3&typeid=10>; 10.1016/j.ijid.2021.09.028 |
|  | Shenzhen | Shenzhen HYK Gene Technology | PSTAR-IIA, SeqExpert III-A | <http://www.hykgene.com/hyk/hyk_platform/index> |
|  | Shenzhen | Anxuyuan Biotechnology (Axbio Inc) | AXP100 | <https://www.axbio.cn/h-col-119.html> |
| Brazil | Campinas | Brazilian Biorenewables National Laboratory (LNBR) | Illumina MiSeq and NextSeq 2000 | <https://lnbr.cnpem.br/facilities/dna-sequencing/> |
|  | Campinas | Universidade Estadual de Campinas (UNICAMP) | Illumina HiSeq 2500 | 10.1007/s00277-020-03986-8 |
|  | Porto Alegre | Universidade Federal do Rio Grande do Sul | Ion Torrent PGM | <https://www.ncbi.nlm.nih.gov/pmc/articles/PMC6687342/> |
|  | Recife | Genomika Diagnósticos | Illumina MiSeq and NextSeq | 10.5151/sbr2019-487 |
|  | Rio de Janeiro | Instituto Oswaldo Cruz | Illumina HiSeq 2500 | <https://www.bjid.org.br/en-next-generation-sequencing-virulome-analysis-yersinia-articulo-S1413867016306237> |
|  | Rio de Janeiro | Bio-Manguinhos/Fiocruz | Illumina MiSeq | <https://doi.org/10.1098/rsfs.2020.0063>; <https://www.ncbi.nlm.nih.gov/pmc/articles/PMC8193464/> |
|  | São Paulo | University of São Paulo | Illumina MiSeq | <https://pubmed.ncbi.nlm.nih.gov/36323834/> |
|  | São Paulo | Universidade Federal de São Paulo | Ion Torrent PGM | <https://journals.lww.com/transplantjournal/Fulltext/2017/12000/Targeted_Next_Generation_Sequencing_in_Brazilian.17.aspx> |
|  | São Paulo | Mendelics | Illumina NovaSeq 6000 | <https://mendelics.com.br/en/technology-and-know-how/> |
|  | São Paulo | DASA S.A. (former Laboratorio Clinico Delboni Auriemo SA) | Illumina NovaSeq 6000 | <https://doi.org/10.21203/rs.3.rs-1641864/v1> |
|  | São Paulo | Hospital Israelita Albert Einstein | Illumina NovaSeq 6000 | <https://www.frontiersin.org/articles/10.3389/fmolb.2022.821582/full> |
| Russia | Moscow | R-pharm | Illumina MiSeq, NovaSeq 6000, iSeq 100, NextSeq 550 | <https://www.r-pharm.com/ru/medical-equipment/oncology> |
|  | Moscow | Genotek | Illumina HiSeq 2500, MiSeq, HiScan; Oxford Nanopore | <https://www.genotek.ru/researches/> |
|  | Moscow | Genoanalytica | Ion PGM; Illumina HiSeq 1500, Thermo Fisher 5500 Genetic Analyzer | <https://www.genoanalytica.ru/seqwithus> |
|  | Moscow | Kurchatov Institute; Kurchatov Genomic Center | DNBSEQ-G400RS, Illumina NovaSeq 6000, MiSeq; Oxford Nanopore GridIon | <https://nrckigc.ru/seque> |
|  | Moscow | Lomonosov Moscow State University | Illumina HiSeq 2000, Illumina MiSeq | <https://sites.google.com/site/evolgenom/materialno-tehniceskaa-baza?pli=1>; <http://algology.ru/1505> |
|  | Moscow | Scientific Research Institute for Systems Biology and Medicine of Rospotrebnadzor | Oxford Nanopore GridION and PromethION, DNBSEQ-G400 | <https://sysbiomed.ru/laboratories/laboratoriya-multiomiksnykh-issledovaniy/> |
|  | Moscow | Lopukhin Federal Research and clinical center of physical-chemical medicine of Federal medical biological agency | Oxford Nanopore;  Illumina HiSeq 2500; Ion Proton | <http://rcpcm.org/nauchnye-issledovanija/centr-kollektivnogo-polzovaniia/> |
|  | Moscow | Evrogen | Illumina NovaSeq 6000, MiSeq | <https://evrogen.ru/services/sequencing/ngs> |
|  | Moscow | Skolkovo Institute of Science and Technology | Illumina HiSeq 4000, MiniSeq | <https://www.skoltech.ru/research/tsentr_genomiki/> |
|  | Moscow | Moscow Institute of Physics and Technology | Illumina MiSeq | <https://genetics.mipt.ru/> |
|  | Astrakhan | Astrakhan State Medical University | Oxford Nanopore MinION | <http://astgmu.ru/nauchno-issledovatelskij-tsentr/sobytiya/> |
|  | Tambov | Derzhavin Tambov State University | Oxford Nanopore MinION | <https://www.tsutmb.ru/about/str/nii/molekulyarno_geneticheskix_issledovanij/> |
|  | Rostov-on-Don | Southern Federal University, CCU «High Technology» | Illumina MiSeq | <http://ckpvt.ru/ru/tskp/naibolee-tsennoe-oborudovanie> |
|  | Kemerovo | Kuzbass State Agricultural Academy | Illumina MiSeq | <https://www.ksai.ru/content/447/nauchnye-laboratorii/centry-kollektivnogo-polzovanija/centr-kollektivnogo-polzovanija-nauchnym-oborudovaniem-v-oblasti-selekcii-i-genetiki-v-zhivotnovodstve/perechen-oborudovanija-ckp.html> |
|  | Tomsk | Tomsk National Research Medical Center | Illumina MiniSeq, MiSeq, NextSeq 2000 | <https://www.tnimc.ru/ckp/oborudovanie/> |
|  | Saint Petersburg | Almazov National Medical Research Center | Illumina MiSeq | <http://www.almazovcentre.ru/?page_id=69710> |
|  | Saint Petersburg | Saint Petersburg State University. Research Park. | Illumina HiSeq 2500 | <https://researchpark.spbu.ru/equipment-biobank-rus/equipment-biobank-genom-rus/equipment-biobank-ngsseq-rus/1762-biobank-hiseq-2500-sequencing-system-rus> |
|  | Volgograd | Volgograd State Medical University | Ion Torrent PGM | <https://ncils.ru/services/otdel-personalizirovannoy-meditsiny-i-klinicheskoy-farmakologii-nauchnogo-tsentra-innovatsionnykh-le/laboratoriya-genomnykh-i-postgenomnykh-tekhnologiy-i-geneticheskogo-kontrolya/> |
|  | Kaliningrad | Immanuel Kant Baltic Federal University | Ion Torrent PGM, Illumina MiSeq | <http://skpntp.kantiana.ru/page4613245.html> |
|  | Kazan | Kazan Federal University | Ion Torrent PGM; Ion Proton; SOLiD xl 5500 Wildfire; Illumina NextSeq 500 | <https://kpfu.ru/science/centry-kollektivnogo-dostupa/mezhdisciplinarnyj-centr-kollektivnogo-polzovaniya/struktura/mezhdisciplinarnyj-centr-proteomnyh-issledovanij/oborudovanie-i-metodiki> |
|  | Kazan | Kazan Institute of Biochemistry and Biophysics of Kazan Science Centre of Russian academy of science | Illumina MiSeq | <http://www.kibb.knc.ru/index.php/ru/equipment> |
|  | Novosibirsk | SB RAS Genomics Core Facility (ICBFM SB RAS) | BGI MGISeq 2000;Illumina MiSeq; Oxford Nanopore MinIon | <http://www.niboch.nsc.ru/doku.php/sequest/instruments> |
|  | Novosibirsk | Siberian Branch of the Academy of Medical Sciences | Ion Torrent PGM | <http://soramn.ru/ckp/?id=1012> |
|  | Yakutsk | North Eastern Federal University | Illumina MiSeq | <https://www.s-vfu.ru/universitet/innovatsii/tsentr-kollektivnogo-polzovaniya/> |
|  | Irkutsk | Federal State Budget Scientific Institution Scientific Centre for Family Health and Human Reproduction Problems (FSBSI SC FHHRP) | Illumina NextSeq 550 | <https://health-family.ru/ru/about-us/center-for-collective-use/> |
|  | Sirius | Sirius University of Science and Technology | Illumina NovaSeq 6000, MiSeq, Oxford Nanopore GridION, MinION | <https://siriusuniversity.ru/research/research-center-for-genetics-and-life-sciences/genomics> |
|  | Vladivostok | Far Eastern Federal University | Ion Torrent S5 | <https://www.dvfu.ru/science/scientific-infrastructure/centers-for-collective-use/equipment/> |
| Kazakhstan | Astana | Nazarbayev University | Illumina NovaSeq6000; Oxford Nanopore PromethION | https://nla.nu.edu.kz/ru/laboratoriya-genomnoj-i-personalizir-3/2 |
| Turkey | Ankara | Sentebiolab | Ion Torrent PGM; Illumina Miseq | https://sentebiolab.com.tr/en/next-generation-sequencing/ |
|  | Ankara | Genomik Araştırmalar | Illumina NovaSeq 6000, iSeq100; Ion Torrent S5; Oxford Nanopore GridION | https://www.agrigx.com/next-generation-sequence-unit-c-1882#prettyPhoto |
|  | Istanbul | İnvitrotek | DNBSEQ-T7, DNBSEQ-G400, DNBSEQ-G50, DNBSEQ-G400 FAST | https://invitrotek.com.tr/kategori/mgi/?l=en |
| Ukraine | Kyiv | LifeCode | Ion Proton | <https://lifecode.com.ua/pro-kompaniyu> |
|  | Kyiv | UltraGenom | Ion Torrent S5 | https://ultragenom.com/ru/laboratoryia/ |
| Belarus | Minsk | Gravita family clinic | Illumina MiSeq | <https://gravita.by/issledovaniya-ploda/preimp-pgt-a/> |
|  | Minsk | Belarusian Research Center for Pediatric Oncology, Hematology and Immunology | Illumina MiSeq | <https://oncology.by/struktura-czentra/nauka/лаборатория-молекулярно-генетических-исследований.html> |
|  | Minsk | Institute of genetics and cytology of the National academy of sciences of Belarus | Illumina MiSeq | <https://igc.by/ru/2016/08/18/genom-ru/> |
| Belgium | Charleroi | DNAVision | Illumina MiSeq, NextSeq | <https://www.dnavision.com/technologies> |
|  | Brussels | BRIGHTcore, Université Libre de Bruxelles | Illumina HiSeq, MiSeq, iScan; Ion Torrent PGM | <https://www.ulb-vub.be/nl/bright-core> |
|  | Ghent | NXTGNT, Ghent University | Illumina MiSeq, NextSeq 500, HiSeq 3000, Novaseq 6000; Oxford Nanopore PromethION | <https://nxtgnt.ugent.be/equipment/> |
|  | Ghent | Molecular Diagnostics UZ Ghent (MDG) | Illumina MiSeq | <https://www.crig.ugent.be/en/network/molecular-diagnostics-uz-ghent-mdg> |
|  | Leuven | KU Leuven Genomics Core, Katholieke Universteit Leuven | Illumina Miseq, NextSeq 500, Hiseq 4000, NovaSeq 6000; PacBio Sequel IIe; Oxford Nanopore PromethION | <https://www.genomicscore.be/instruments_services-1/ngs-instruments/> |
|  | Leuven | VIB-KU Leuven Center for Cancer Biology (CCB) | Illumina iSCAN, HiSeq 2000 | <https://ccb.sites.vib.be/en/expertise/genetic-technologies> |
|  | Liège | GIGA Genomics, Liège University | Illumina 2x iScan, 2x MiSeq, NextSeq 500, NextSeq 550, NovaSeq 6000; Oxford Nanopore - MinION | <https://www.gigagenomics.uliege.be/cms/c_4353484/en/gigagenomics-equipment> |
|  | Antwerp | Laboratory of Medical Microbiology, University of Antwerp | Illumina MiSeq; PacBio Sequel; Oxford Nanopore MinION | <https://www.uantwerpen.be/en/research-groups/laboratory-of-medical-microbiology/our-services/next-generation-sequ/> |
|  | Antwerp | Neuromics Support Facility,  University of Antwerp | Illumina MiSeq, NextSeq500 | <https://www.neuromicssupportfacility.be/service/sequencing> |
|  | Antwerp | GENDIA | Illumina NextSeq, HiSeq 4000 | <https://www.gendia.eu/whole-exome-sequencing-wes/> |
| Austria | Wien | Vienna BioCenter Core Facilities | Illumina MiSeq, NextSeq, NovaSeq; PacBio Sequel II; PromethION; MinION | <https://www.viennabiocenter.org/vbcf/next-generation-sequencing/> |
|  | Wien | AGES | Illumina, Oxford Nanopore | <https://www.ages.at/en/ages/events/laboratory-practice/next-generation-sequencing-ngs?sword_list%5B0%5D=illumina&no_cache=1> |
|  | Wien | CEMM The Research Center for Molecular Medicine of the Austrian Academy of Sciences | Illumina HiSeq | <https://cemm.at/research/topics/post-genomic-technologies> |
| Finland | Helsinki | University of Helsinki (Finland – Institute for Molecular Medicine) | Illumina NovaSeq, NextSeq500, MiSeq, iSeq;  PacBio Sequel II | <https://hilife-infra.ilab.agilent.com/service_center/show_external/3612?name=dna-sequencing-and-genomics-laboratory-bidgen> |
|  | Helsinki | Laboratory of DNA Sequencing and Genomics (BIDGEN), University of Helsinki | Illumina MiSeq, NextSeq 500, NovaSeq 6000; PacBio Sequel II | <https://www.helsinki.fi/en/researchgroups/dna-sequencing-and-genomics/dna-sequencing-service> |
|  | Turku | Turku Bioscience | Illumina MiSeq, NovaSeq 6000 | <https://bioscience.fi/services/functional-genomics/instruments/> |
|  | Tampere | Tampere University | Illumina Miseq | <https://www.tuni.fi/en/research/tampere-genomics-facility> |
| Latvia | Riga | Latvian Biomedical research and study center | Illumina MiSeq; DNBSEQ-G400, DNBSEQ-T7 | <https://biomed.lu.lv/services/core-facility/genome-centre-2/> |
| Germany | Stuttgart | Fraunhofer IGB | Illumina HiSeq 2500 | <https://www.igb.fraunhofer.de/en/research/in-vitro-diagnostics/services.html> |
|  | Köln | Cologne Center for Genomics | Illumina 2x Genome Analyzer llx, NovaSeq 6000; Roche 454 FLX Titanium; Oxford Nanopore GridION | <https://www.nucleics.com/dna-sequencing-support/dna-sequencing-service-reviews/cologne-center-of-genomics+>; <https://wggc.de/wp-content/uploads/2020/11/020_Flyer_WGGC_final_web.pdf> |
|  | Bonn | NGS core facility Uniklinik Bonn | Illumina NovaSeq 6000, NextSeq 550, MiSeq, MiniSeq, iSeq 100 | <https://btc.uni-bonn.de/ngs/first-visit/> |
|  | Düsseldorf | Heinrich Heine University Düsseldorf | Illumina HiSeq 3000, MiSeq; Ion Torrent PGM, Ion S5 and Proton; PacBio Sequel I and II; Bionano Saphyr; Oxford Nanopore GridION, PromethION | <https://www.gtl.hhu.de/en.html> |
|  | Dresden | Max Planck Institute of Molecular Cell Biology and Genetics | PacBio Sequel I and II; Oxford Nanopore PromethION | <https://www.mpi-cbg.de/research/scientific-cores-support/scientific-services/sequencing-and-genotyping/equipment> |
|  | Dresden | Technische Universität Dresden | PacBio Sequel; Illumina NovaSeq 6000,  HiSeq 2500,  NextSeq 500,  MiSeq;  Bionano Saphyr;  Oxford Nanopore PromethION | <https://tu-dresden.de/cmcb/technologie-plattform/facilities/deep-sequencing/geraete> |
|  | Kiel | Competence Centre for Genomic Analysis (CCGA) Kiel | Illumina 2x NovaSeq 6000, HiSeq 4000, NextSeq 500, 3x MiSeq; PacBio Sequel II | <https://ccga.uni-kiel.de/landing_page/about_us> |
|  | Tübingen | NGS Competence Center Tübingen (NCCT) | Illumina NextSeq 500, NovaSeq 6000, MiSeq; PacBio Sequel II; Oxford Nanopore MinION, PromethION | <https://portal.qbic.uni-tuebingen.de/portal/web/ncct/sequencing-solutions> |
|  | Tübingen | CeGaT | Illumina NovaSeq 6000 | <https://www.cegat.com/services/genome-sequencing/> |
|  | München | Ludwig-Maximilians-Universität München | Illumina NextSeq 1000, NextSeq 2000; Oxford Nanopore MinION, PromethION | <https://www.genzentrum.uni-muenchen.de/research-groups/lafuga/blum/technologies/index.html> |
|  | Berlin | Berlin Institute of Health | Illumina MiSeq, MiniSeq, iSeq, NextSeq 500, HiSeq 6000; PacBio Sequel II; Oxford Nanopore MinION, PromethION | <https://www.bihealth.org/de/forschung/wissenschaftliche-infrastruktur/core-facilities/genomik> |
|  | Leipzig | Max Planck Institute for Evolutionary Anthropology | Illumina HiSeq 2500, MiSeq; PacBio Sequel II | <https://www.eva.mpg.de/genetics/laboratory-facilities/> |
|  | Leipzig | Genewiz | Illumina NovaSeq 6000, HiSeq X, MiSeq; PacBio Sequel | <https://www.genewiz.com/en-gb/Public/Resources/NGS-Platforms> |
|  | Leipzig | PathoNext GmbH | Illumina MiSeq, NextSeq 500 and NovaSeq 6000 | <https://www.pathonext.de/leistungen/sequenzierungen> |
|  | Leipzig | Universität Leipzig | Illumina HiScan SQ; IonTorrent PGM | <https://home.uni-leipzig.de/izkf/dna/nextgenseq.html> |
| United Kingdom | Surrey | Medical testing laboratory "Igenomix" | Ion Torrent S5 | <https://www.igenomix.co.uk/> |
|  | Lexington | UK HealthCare Genomics Core Laboratory | NovaSeq 6000, NextSeq 2000, MiSeq | <https://ukhealthcare.uky.edu/> |
|  | Cambridge | Cambridge Genomic Services | Illumina и Oxford Nanopore | <https://www.cam.ac.uk/> |
|  |  | The National Health Service England | Illumina MiSeq, Ion Torrent S5 | <https://www.nhs.uk/> |
|  | Liverpool | University of Liverpool | Illumina NovaSeq 6000, PacBio Sequel II | <https://www.liverpool.ac.uk/genomic-research/technologies/next-generation-sequencing/> |
|  | Cambridge | Novogene (UK) Company Limited | Illumina NovaSeq 6000; Oxford Nanopore PromethION, PacBio Sequel II/IIe | <https://www.novogene.com/eu-en/services/research-services/genome-sequencing/> |
|  | Leeds | University of Leeds (Next Generation Sequencing Facility) | Illumina HiSeq 3000, NextSeq, MiSeq; PacBio Sequel; Oxford Nanopore Minions | <https://medicinehealth.leeds.ac.uk/> |
|  | Oxford | Department of Biochemistry, Dorothy Crowfoot Hodgkin Building, University of Oxford | Illumina, PacBio Sequel IIe | <https://www.ox.ac.uk/> |
|  | London | The London Regional Genomics Centre | Ion Torrent PGM, Illumina MiSeq, NextSeq | <https://www.robarts.ca/lrgc/> |
|  | London | Imperial BRC Genomics Facility | Illumina HiSeq 4000, MiSeq | <https://www.imperial.ac.uk/medicine/research-and-impact/facilities/genomics-facility/> |
|  | Bristol | Bristol Genomics Facility, University of Bristol | Illumina MiSeq, NextSeq 500 | <https://www.bristol.ac.uk/> |
| Spain | Barcelona | PALEX MEDICAL S.A. | Ion Torrent S5 | <https://www.rafer.es/> |
|  | Barcelona | Quantitative Genomic Medicine Laboratories, SL. | Illumina NextSeq | <https://qgenomics.com/servicios/secuenciacion-ngs-old/> |
|  | Madrid | Centro Nacional de Investigaciones Cardiovasculares Carlos III (F.S.P.) | Illumina NextSeq 2000, MiSeq, NovaSeq 6000; Oxford Nanopore MinION | <https://www.cnic.es/es/investigacion/2/795/tecnologia> |
|  | Madrid | Instituto Ramón y Cajal de Investigación Sanitaria | Illumina MiSeq | <https://www.irycis.org/en/> |
|  | Barcelona | The Hospital Clínic de Barcelona | Illumina MiSeq, NextSeq 2000 | <https://www.clinicbarcelona.org/en/idibaps> |
|  | Reus | Centre for Omic Sciences | Ion Torrent PGM | <https://omicscentre.com/services-applications/genomics-and-transcriptomics/next-generation-sequencing-ion-torrent-pgm/> |
|  | Valencia | INCLIVA – Instituto de Investigación Sanitaria | Illumina NextSeq 550 | <https://www.incliva.es/> |
| The Netherlands | Amsterdam | Macrogen Europe | Illumina NovaSeq 6000, HiSeq X, HiSeq 2500, NextSeq 500, MiSeq; PacBio Sequel II | <https://www.macrogen-europe.com/> |
|  | Amsterdam | Amsterdam University Medical Center | Illumina, Ion Torrent, PacBio, Oxford Nanopore | <https://www.amsterdamumc.org/en/research/core-facility/genomics/techniques/next-generation-sequencing.htm> |
|  | Amsterdam | Swammerdam Institute for Life Sciences, University of Amsterdam | Illumina NextSeq 550; Ion Proton | <http://www.rnabiology.nl/rnabiology.html> |
|  | Amsterdam | Netherlands Cancer Institute | Illumina NovaSeq 6000, HiSeq 2500, NextSeq 550 and MiSeq | <https://www.nki.nl/research/facilities-platforms/genomics-core-facility/> |
|  | Amsterdam | Hartwig Sequencing Services | 3x Illumina NovaSeq 6000 | <https://hartwigsequencingservices.nl/technology/> |
|  | Rotterdam | Genomics Core Facility (Erasmus Universitair Medisch Centrum) | Illumina HiSeq 4000, HiSeq 2500, MiSeq | <https://www.dtls.nl/technology-hotels/list/rotterdam-genomics-core/> |
|  | Maastricht | Maastricht University Medical Centre | Illumina NextSeq, NovaSeq;  PacBio Sequel I and II; BioNano Saphire | <https://klinischegenetica.mumc.nl/genome-services-maastricht-umc/genomics> |
|  | Utrecht | Utrecht University | Illumina NextSeq500, HiSeq2500, MiSeq and MiniSeq | <https://www.uu.nl/en/research/life-sciences/facilities/facilities-for-molecular-research/utrecht-sequencing-facility> |
|  | Leiden | Leiden University Medical Center | Oxford Nanopore MinION and Flongle; PacBio Sequel II | <https://www.lumc.nl/en/research/facilities/leiden-genome-technology-center-lgtc/equipment/> |
|  | Leiden | GenomeScan | Illumina NovaSeq 6000; PacBio Sequel II; Ion Torrent GeneStudio S5 | <https://www.genomescan.nl/our-sequencing-instruments/> |
|  | Groningen | University of Groningen | Illumina NextSeq 500 и MiSeq | <https://www.rug.nl/> |
| Sweden | Stockholm | SciLifeLab (Science for Life Laboratory) | Illumina NovaSeq 6000, iSeq | <https://www.scilifelab.se/> |
|  | Stockholm, Uppsala | National Genomic Infrastructure (NGI) | Illumina iScan, iSeq 100, MiSeq, NextSeq 2000, NovaSeq 6000; Ion Torrent S5 XL; Oxford Nanopore MinION, PromethION, Flonge | <https://ngisweden.scilifelab.se/technologies/genotyping-systems/> |
|  | Lund | Lund University | Illumina NovaSeq 6000, NextSeq 2000, MiSeq, NextSeq 500 | <https://www.ctg.lu.se/genomic-services/sequencing-only-0>; <https://portal.research.lu.se/en/equipments/center-for-translational-genomics> |
|  | Lund | MultiPark (Multidisciplinary research focused on Parkinson´s diseas) at Lund University | Illumina NextSeq 500 | <https://www.multipark.lu.se/sites/multipark.lu.se/files/2022-10/NGS_styrdokument_20221019%20%28kopia%29_0.pdf> |
|  | Uppsala | Uppsala University | Illumina NextSeq 550, MiSeq, MiniSeq; Oxford Nanopore GridION​ | <https://www.uu.se/en> |
|  | Gothenburg | University of Gothenburg | 2x Ion Torrent S5 Prime; Illumina 3x MiSeq, NextSeq 550, 2x NovaSeq 6000 | <https://www.gu.se/en/core-facilities/bioinformatics-and-data-centre-bdc/clinical-genomics-gothenburg> |
|  | Uppsala | Swedish University of Agricultural Sciences | Illumina MiSeq | <https://www.slu.se/en/departments/aquatic-sciences-assessment/research/forskningsomraden/Functional-microbial-ecology/> |
|  | Linköping | Linköping University | Illumina MiSeq | <https://liu.se/en/article/core-facility-molekylarbiologi> |
|  | Umeå | Umeå University | Illumina 2x NextSeq 550, MiSeq; Oxford Nanopore MinION | <https://www.umu.se/en/> |
| Denmark | Aarhus | The Department of Molecular Medicine (MOMA) Aarhus University Hospital | Illumina NovaSeq 6000, MiSeq | <https://moma.dk/> |
|  | Copenhagen | University of Copenhagen | Illumina MiSeq, NextSeq 550 | <https://www.ku.dk/english/>; <https://www.bric.ku.dk/core-facilities/sequencing/> |
|  | Aalborg | Aalborg University | 454 Life Sciences, Solexa | <https://vbn.aau.dk/en/> |
|  | Odense | Odense University Hospital | Illumina NovaSeq 6000, NextSeq 550; Oxford Nanopore PromethION | <https://www.sdu.dk/en/forskning/klinisk_genom_center/platforms> |
| Lithuania | Vilnius | Vilnius University | Illumina MiSeq | <https://doi.org/10.1038/s41586-021-04058-1> |
|  | Kaunas | Lithuanian University of Health Sciences | Illumina MiSeq | <https://doi.org/10.3390/medicina58091240> |
| Luxembourg | Luxembourg | LUXGEN Luxembourg Institute of Health | Illumina Miseq, NextSeq 550 | <https://sites.lih.lu/luxgen/technologies/ngs/> |
|  | Luxembourg | Université du Luxembourg | Illumina MiSeq, NextSeq 500; Oxford Nanopore MinION | <https://wwwfr.uni.lu/lcsb/services/platforms/sequencing_platform> |
| Israel | Rehovot | The Nancy and Stephen Grand Israel National Center for personalized medicine (G-INCPM) | Illumina NovaSeq 6000, MiSeq, HiSeq 2500, NextSeq 500; PacBio Sequel | <https://g-incpm.weizmann.ac.il/units/CrownGenomics/equipment> |
|  | Katzrin | GGA Genetic Center | Illumina NextSeq, MiSeq | <https://www.gga.org.il/about-heb?lang=en> |
|  | Jerusalem | Hadassah Medical Center | Illumina NovaSeq 6000 | <https://www.hadassah.org.il/en/exome_sequencing_lab/> |
|  | Jerusalem | The Hebrew University of Jerusalem | Illumina NextSeq 500, MiSeq | <https://www.bio.huji.ac.il/en/units_the_national_center_for_genomic_technologies> |
|  | Haifa | Rambam Health Care Campus | Ion Torrent S5 | <https://doi.org/10.3389/fcimb.2022.955481> |
|  | Tel Aviv | Tel Aviv University | Illumina NextSeq 500, MiSeq | <https://en-lifesci.tau.ac.il/zabam/ngs/ngs> |
| France | Lille | European Diabetes Genomics Institute (EGID) in the Lille University Hospital; Université de Lille | Illumina MiSeq, NextSeq 500, NovaSeq 6000, iSeq100; Oxford Nanopore MinION | <https://pubmed.ncbi.nlm.nih.gov/33046911/> |
|  | Paris | Institute of Biology of the École Normale Supérieure | Illumina NextSeq 2000 and NextSeq 500; Oxford Nanopore MinION and MinION Mk1C | <https://doi.org/10.1016/j.celrep.2020.108076> |
|  | Paris | Institut Curie | Illumina NovaSeq 6000 and MiSeq; PacBio Sequel IIe | <https://www.france-genomique.org/plateformes-et-equipements/> |
|  | Paris | Institut Pasteur | Illumina NextSeq 2000, NextSeq 500, MiniSeq, ISeq100, MiSeq; PacBio Sequel; Oxford Nanopore Minion | <https://www.france-genomique.org/plateformes-et-equipements/> |
|  | Paris | Cochin Institute | Illumina Nextseq 500, MiSeq; Ion Torrent PGM, S5XL | <https://www.france-genomique.org/plateformes-et-equipements/> |
|  | Paris | Paris Brain Institute | Illumina Novaseq 6000, Nextseq 500, MiSeq |  |
|  | Fontenay-aux-Roses | François Jacob Institute Of Biology | Illumina NovaSeq, MiSeq; Oxford Nanopore MinION, GridION, PromethION; MGI DNBSEQ-G400; Bionano Saphyr |  |
|  | Lyon | Université de Lyon | Illumina MiSeq, NextSeq 500 and HiSeq 2500; Oxford Nanopore MinION |  |
|  | Nice | Université de Nice Sophia-Antipolis | Illumina NextSeq 500; Oxford Nanopore MinION and PromethION |  |
|  | Marseille | Aix-Marseille University | Illumina NextSeq 500 |  |
|  | Montpellier | MGX-Montpellier GenomiX | Illumina NovaSeq 6000 and MiniSeq; Oxford Nanopore MinION | <https://www.mgx.cnrs.fr/> |
|  | Toulouse | GénoToul | Illumina NovaSeq, 3x Miseq, iSeq100; 2x Sequel II Pac Bio; Oxford Nanopore 2x MinION, GridION, PromethION; Ion Torrent S5 | <https://get.genotoul.fr/portfolio_page/sequencage-nouvelle-generation/> |
|  | Bordeaux | La Plateforme Génome Transcriptome de Bordeaux (PGTB) | Illumina NextSeq 2000, MiSeq, iSeq 100; Oxford Nanopore GridION, MinION Mk1C | <https://pgtb.fr/equipements/> |
|  | Clermont-Ferrand | Gentyane | PacBio 2x Sequel II; Bionano Saphyr; Thermo AgriSeq; Ion Torrent S5 | <https://gentyane.clermont.inrae.fr/contenu/equipements> |
|  | Nantes | GenoA (Genomics Atlantic) | Illumina NovaSeq 6000, MiSeq | <https://pf-genomique.univ-nantes.fr/en/equipments> |
|  | Gif-Sur-Yvette | Institut de Biologie Intégrative de la Cellule (I2BC) | Illumina NextSeq 550 and NextSeq 500; Oxford Nanopore GridION | <https://www.i2bc.paris-saclay.fr/sequencing/ng-sequencing/addon-ng-sequencing/#equipements> |
|  | Rennes | Université de Rennes | Illumina 2x MiSeq; DNBSEQ-G400 | <https://www.france-genomique.org/plateformes-et-equipements/plateforme-sequencage-geh-rennes/>; <https://osur.univ-rennes.fr/ecogeno-transcriptomique-eng#p-2021> |
| Italy | Turin | University of Turin | Illumina NextSeq 500, NextSeq 1000, MiSeq | <https://dbiosen.campusnet.unito.it/do/home.pl/View?doc=/servizi_esterno/Genomic_platform.html> |
|  | Turin | Italian Institute for Genomic Medicine | Illumina iScan, NovaSeq 6000; Oxford Nanopore GridION | <https://www.iigm.it/site/index.php?id=142&t=articolo> |
|  | Genova | Istituto Italiano di Tecnologia | Illumina Novaseq 6000, HiSeq 2000 and MiSeq; Oxford Nanopore GridIon | <https://genomics.iit.it/technological-facilities> |
|  | Milan | IRCCS Ospedale San Raffaele | Illumina HiSeq 2500, Novaseq 6000, NextSeq 500, MiSeq; Oxford Nanopore MinIon | <https://research.hsr.it/en/centers/omics-sciences/ngs.html> |
|  | Milan | Labospace Srl | Ion Torrent S5 | <https://www.labospace.com/microbioma/> |
|  | Milan | Human  Technopole | Illumina NovaSeq 6000, MiSeq; Oxford Nanopore PromethION | <https://humantechnopole.it/en/facilities/genomics-facility/> |
|  | Milan | Istituto Oncologico Europeo | Ion Torrent S5 | <https://doi.org/10.3389/fonc.2020.00236> |
|  | Milan | IRCCS Istituto Auxologico Italiano | Illumina NextSeq 500 | 10.3389/fendo.2021.664645 |
|  | Foggia | Casa Sollievo della Sofferenza | Illumina NextSeq 500, MiSeq; Ion Torrent S5 | <https://www.alleanzacontroilcancro.it/istituto/irccs-casa-sollievo-della-sofferenza/> |
|  | Trento | University of Trento | lllumina NovaSeq 6000, and MiSeq; Oxford Nanopore MinION | <https://www.cibio.unitn.it/50/next-generation-sequencing-ngs> |
|  | Bologna | Universita di Bologna | Illumina MiSeq | <https://dimes.unibo.it/en/research/research-laboratories/diagnostic-area/microbiology-laboratory-cesena-campus> |
|  | Florence | Università degli Studi di Firenze | Ion Torrent PGM; Illumina Novaseq 6000; PacBio Sequel | <https://www.ibbr.cnr.it/ibbr/facilities/genomic-lab>; <https://endemixit.com/2020/05/25/meet-our-sequencing-platforms-at-the-university-of-florence/> |
|  | Rome | University La Sapienza – Rome (DMM) | Ion Torrent S5Xl | <https://doi.org/10.3389/fonc.2020.00236> |
|  | Bari | Tumori Giovanni Paolo II | Ion Torrent PGM | <https://doi.org/10.3389/fonc.2020.00236> |
|  | Sassari | Consiglio Nazionale delle Ricerche | Ion Torrent PGM | <https://doi.org/10.3389/fonc.2020.00236> |
|  | Meldola | Istituto Scientifico Romagnolo per lo Studio e la Cura dei Tumori (IRST) IRCCS | Illumina MiSeq | <https://doi.org/10.18632/oncotarget.16791> |
|  | Naples | University of Naples Federico II | Ion Torrent PGM | <https://doi.org/10.3389/fonc.2020.00236> |
| Poland | Warszawa | Genomed SA | Illumina MiSeq; Ion Proton; Oxford Nanopore MinION | <http://www.genomed.pl/index.php/en/technology> |
|  | Warszawa | Centre of New Technologies University of Warsaw | Illumina NovaSeq 6000, NextSeq 500, MiSeq | <https://cent.uw.edu.pl/en/core-facilities/genomics-core-facility/#menu2> |
|  | Wrocław, Złotniki | genXone SA | Oxford Nanopore PromethION, GridION | <https://genxone.eu/en/technology/> |
|  | Wrocław | Instytut Immunologii i Terapii Doświadczalnej im. Ludwika Hirszfelda | Illumina MiSeq, NextSeq 550; Oxford Nanopore MinIon | <https://binwit.pl/sekwencjonowanie-nastepnej-generacji-z-ang-next-generation-sequencing-ngs/> |
|  | Bydgoszcz | Centrum Onkologii im. Prof. F. Łukaszczyk | Illumina MiniSeq and NextSeq 550 | <https://co.bydgoszcz.pl/en/diagnostic-and-treatment-plants/department-of-genetics-and-molecular-oncology-2/> |
|  | Kraków | Jagiellonian University Medical College | Illumina MiSeq, Roche GS Junior | <https://omicron.cm.uj.edu.pl/en/techniques/equipment/> |
|  | Gdańsk | Fahrenheit Universities | Illumina MiSeq | <https://resources.faru.edu.pl/infrastructure/876/next-generation-sequencing-platform> |
|  | Poznan | Poznan University of Medical Sciences | Ion Torrent | <https://uca.ump.edu.pl/en/laboratories/> |
|  | Lodz | Medical University of Lodz | Illumina NextSeq 550 | <https://fixnet.umed.pl/knowledge/ngs/> |
| Greece | Athens | Greek Genome Center | Illumina NovaSeq 6000, NextSeq 500, MiSeq | <http://genome.bioacademy.gr/equipment/#equipment> |
|  | Athens | Institute of Marine Biology, Biotechnology and Aquaculture (IMBBC) | Roche/454 GS FLX; Illumina MiSeq; Oxford Nanopore MinION | <https://imbbc.hcmr.gr/infrastructures/facilitiesimbbc/dna-sequencing-platform/> |
|  | Patras | University of Patras | Oxford Nanopore MinION | <https://2021.igem.org/Team:Patras/Engineering> |
| Switzerland | Bern | University of Bern | Illumina NovaSeq 6000, NextSeq 1000, MiSeq, iSeq 100;  PacBio Sequel IIe | <https://www.ngs.unibe.ch/technology/> |
|  | Zürich | Eidgenössische Technische Hochschule Zürich | Illumina HiSeq 2500, NextSeq 500, MiSeq | <https://bsse.ethz.ch/genomicsbasel/equipment/next-generation-sequencing.html> |
|  | Lausanne | Swiss Federal Institute of Technology Lausanne (EPFL) | Illumina NextSeq 500, NovaSeq 6000 and MiSeq; Oxford Nanopore MinION | <https://www.epfl.ch/research/facilities/gene-expression-core-facility/next-generation-sequencing/> |
|  | Lausanne | Lausanne Genomic Technologies Facility | Illumina NovaSeq 6000 and MiSeq; PacBio Sequel II | <https://wp.unil.ch/gtf/technologies/> |
|  | Basel | Friedrich Miescher Institute for Biomedical Research | MiSeq, NextSeq and HiSeq2500 | <https://wp.unil.ch/gtf/technologies/> |
|  | Geneva | Genomic Research Laboratory of Geneva University Hospital | Illumina-Solexa Genome Analyzer II | <https://www.genomic.ch/technology.php> |
| Norway | Oslo | Norwegian Sequencing Centre | Illumina Novaseq 6000, NextSeq 500, MiSeq; PacBio Sequel IIe | <https://www.sequencing.uio.no/> |
|  | Bergen | University of Bergen | Illumina NovaSeq 6000, NextSeq 500, MiSeq | <https://www.uib.no/en/clin2/genomics/94652/high-throughput-sequencing> |
|  | Tromsø | UiT The Arctic University of Norway | Illumina NextSeq 550; Oxford Nanopore GridION | <https://uit.no/prosjekter/prosjektsub?p_document_id=468291&sub_id=468396> |
|  | Trondheim | Norwegian University of Science and Technology | Illumina NovaSeq 6000 and NextSeq 500 | <https://www.ntnu.edu/mh/gcf/services-and-equipment> |
| Iceland | Reykjavík | University of Iceland | Illumina Miseq | <http://lifvisindi.hi.is/core-facilities> |
| Estonia | Tartu | University of Tartu | Illumina HiSeq 2500, MiSeq, NextSeq 500, iScan | <https://cgem.ut.ee/facilities>; <https://genomics.ut.ee/en/next-generation-sequencing> |
| Portugal | Caparica | STAB VIDA, Lda. | Ion Torrent, Illumina HiSeq 2500 and MiSeq | <https://www.stabvida.com/next-generation-sequencing-of-dna> |
|  | Alverca do Ribatejo | Quimigen Portugal | Illumina; Roche 454; Ion torrent: Proton, PGM | <https://www.quimigen.pt/buy/cat-next-generation-sequencing-ngs-3452.html> |
| Korea | Seoul | Macrogen | Illumina HiSeq 2500, HiSeq 4000, HiSeq X Ten, NovaSeq 6000, NextSeq 500, MiSeq; PacBio Sequel I and II; Oxford Nanopore PromethION | <https://www.macrogen.com/en/business/research/ngs> |
|  | Seoul | DNA Link | NovaSeq 6000, NextSeq 500, MiSeq; PacBio Sequel, Sequel IIe; Oxford Nanopore MinION | <https://dnalink.com/ngs> |
|  | Suwon | Theragen Bio | Illumina NovaSeq 6000, MiSeq; PacBio Sequel Ⅱ | <https://www.theragenbio.com/eng/service/ngs01_1.php?category=ngs01_1> |
| Japan | Tokyo | National Cancer Center of Japan | Illumina NextSeq 550 | <https://www.ncc.go.jp/en/information/press_release/20180403_2/index.html>  <https://www.illumina.com/company/news-center/feature-articles/illumina-registered-nextseq-550dx-instrument-in-japan.html> |
|  | Tokyo | Riken | Illumina NovaSeq 6000, HiSeq, NextSeq, MiSeq; Ion Torrent S5 | <https://www.rikengenesis.jp/service_db/category01/item_14> |
|  | Kyoto | Kyoto University | Oxford Nanopore PromethION; Illumina NovaSeq 6000, NextSeq 550, iSeq 100 | <https://ashbi.kyoto-u.ac.jp/core-facility/guide/> |
|  | Akita | Akita Prefectural University | Illumina HiSeq 1000 | <https://doi.org/10.3390/molecules22122270> |
|  | Hokkaido | Hokkaido System Science | Illumina HiSeq 2000, HiSeq 2500 | <https://doi.org/10.1128/mra.00472-22>; <https://doi.org/10.3389/fpls.2022.850660> |
| Australia | Melbourne | AGRF | Illumina NovaSeq 6000 | <https://www.agrf.org.au/next-generation-sequencing> |
|  | Melbourne | WEHI—Walter and Eliza Hall Institute of Medical Research | Illumina MiSeq and NextSeq 500; Oxford Nanopore PromethION; MGI; PacBio Sequel | <https://www.wehi.edu.au/people/rory-bowden/4536/wehi-advanced-genomics-facility> |
|  | Sydney | Garvan Institute of Medical Research | Oxford Nanopore PromethION and GridION | <https://www.garvan.org.au/research/kinghorn-centre-for-clinical-genomics/research-programs/genomic-technologies> |
|  | Melbourne | Deakin Genomics Centre | Illumina NovaSeq 6000, MiniSeq, MiSeq; Oxford Nanopore | <https://www.deakin.edu.au/life-environmental-sciences/facilities/deakin-genomics-centre> |
|  | Melbourne | The Doherty Institute | Illumina NextSeq, Next Gen 454; Ion Torrent | <https://www.doherty.edu.au/services-facilities/facilities> |
|  | Sydney | Western Sydney University | Illumina NovaSeq 6000, Illumina MiSeq | <https://www.westernsydney.edu.au/research/centralised_research_facilities/next_generation_genome_sequencing_facility> |
| New Zealand | Palmerston North | Massey University | 2x Illumina MiSeq | <https://www.massey.ac.nz/about/clinics-and-services-for-the-public/massey-genome-service/next-generation-sequencing-ngs/> |
|  | Auckland | The University of Auckland | Illumina MiSeq, HiSeq, and NovaSeq; Oxford Nanopore PromethION, GridION, MinIONs, MK1Cs, and flongles | [https://www.auckland.ac.nz/en/science/about-the-faculty/share-shared-research-equipment/auckland- genomics.html](https://www.auckland.ac.nz/en/science/about-the-faculty/share-shared-research-equipment/auckland-%20genomics.html) |
|  | Dunedin | The University of Otago | Illumina MiSeq, NextSeq 2000; Oxford Nanopore GridION | <https://www.otago.ac.nz/genomics/index.html> |
|  | Wellington | Institute of Environmental Science and Research (ESR) | Oxford Nanopore GridION and MinION | <https://www.esr.cri.nz/our-services/consultancy/genomics-and-next-generation-sequencing/> |
|  | Wellington | Wellington Regional Genetics Laboratory | Illumina iSeq 100 | <http://www.wellingtongenetics.co.nz/shop/Molecular+Genetic+%28DNA%29+Test+List/NGS+Cancer+Hotspot+Panel.html> |
| USA | Seattle, WA | Northwest Genomics Center; University of Washington | Illumina MiSeq, NextSeq 500, NovaSeq 6000 | https://nwgc.gs.washington.edu/?q=what-we-do/equipment |
|  | Seattle, WA | Fred Hutchinson Cancer Center | Illumina 3x MiSeq, 3x NextSeq 2000, NovaSeq 6000; PacBio Sequel IIe | https://www.fredhutch.org/en/research/shared-resources/core-facilities/genomics-bioinformatics/genomics-services.html#services |
|  | Seattle, WA | Institute for Systems Biology | Illumina NextSeq | https://corefacilities.isbscience.org/genomics/next-gen-sequencing/ |
|  | San Francisco, CA | UCSF Institute for Human Genetics; University of California San Francisco | Illumina 2x MiniSeq, MiSeq, HiSeq 2500, HiSeq 4000, 2x NovaSeq 6000 | <https://humangenetics.ucsf.edu/services#Illumina-Sequencing>  <https://pcat.ucsf.edu/equipment>  https://uccore.org/ucsf-center-for-advanced-technology/ |
|  | Berkeley, CA | University of California, Berkeley | Illumina iSeq 100, MiSeq, NextSeq 2000, HiSeq 4000, NovaSeq 6000; PacBio Sequel II; Oxford Nanopore MinION | <https://innovativegenomics.org/ngs-core/>; https://qb3.berkeley.edu/facility/genomics/ |
|  | Davis, CA | DNA Technologies Core; University of California Davis | Illumina 3x MiSeq, NextSeq 500, NovaSeq 6000; PacBio Sequel II, Sequel IIe; Oxford Nanopore PromethION; Element Biosciences Aviti; Bionano Saphyr | https://dnatech.genomecenter.ucdavis.edu/ |
|  | Sacramento, CA | Comprehensive Cancer Center; University of California Davis | Illumina MiSeq | https://health.ucdavis.edu/cancer/research/sharedresources/im/equipment-immune.html |
|  | Fremont, CA | SeqMatic | Illumina MiSeq, NextSeq 500, NovaSeq 6000 | https://www.seqmatic.com/about/# |
|  | Palo Alto, CA | Stanford Center for Genomics and Personalized Medicine | llumina MiSeq, HiSeq 4000, HiSeq 2500; PacBio Sequel | https://med.stanford.edu/gssc.html |
|  | Salt Lake City, UT | University of Utah Genomics Core | Ion Torrent Proton | <https://cores.utah.edu/dna-sequencing/#1518114083588-fbd34a5a-e2c0>  https://cores.utah.edu/genomics/genomics-whole-genome-snp-genotyping/ |
|  | Bronx, NY | Epigenomics Shared Facility; Albert Einstein College of Medicine | Illumina MiSeq, NextSeq 500, HiSeq 2500; Oxford Nanopore MinION | https://einstein.ilabsolutions.com/service_center/show_external/3976?name=epigenomics-shared-facility |
|  | Salt Lake City, UT | Huntsman Cancer Institute; University of Utah Health Sciences | Illumina 2x MiSeq, 2x HiSeq 2500, NovaSeq 6000 | https://uofuhealth.utah.edu/huntsman/shared-resources/gba/htg |
|  | Provo, UT | DNA Sequencing Center; Brigham Young University | Illumina HiSeq 2500; PacBio Sequel I and Sequel II | https://biology.byu.edu/dnasc/resources |
|  | Logan, UT | Center for Integrated BioSystems; Utah State University | Illumina Miseq, NextSeq | https://caas.usu.edu/biosystems/core-labs/genomics |
|  | Denver, CO | National Jewish Health | Illumina MiSeq; Ion Torrent S5 Prime, Ion S5 XL | https://www.nationaljewish.org/research-science/programs-depts/center-for-genes-environment-and-health/genomics-facility |
|  | Aurora, CO | University of Colorado Anschutz Medical Campus | Illumina iSeq, MiSeq, NovaSeq 2000, NovaSeq 6000 | https://medschool.cuanschutz.edu/colorado-cancer-center/research/shared-resources/genomics/instruments |
|  | Albuquerque, NM | University of New Mexico Health Sciences Center and UNM Comprehensive Cancer Center | Ion Torrent S5 Prime, Ion S5 XL | https://genomics.unm.edu/atg/technologies/sequencing.html |
|  | Albuquerque, NM | University of New Mexico | Illumina NextSeq 500 | https://ceti.unm.edu/core-facilities/molecular-biology.html |
|  | Tempe, AZ | Arizona State University Genomics Facility | Illumina MiSeq, NextSeq 500 | https://cores.research.asu.edu/genomics/equipment |
|  | Phoenix, AZ | Translational Genomics Research Institute (TGen) | Illumina MiSeq, NextSeq, HiSeq, NovaSeq | https://www.tgen.org/research/shared-resources-services/collaborative-sequencing-center/ |
|  | Tucson, AZ | Research, Innovation and Impact; The University of Arizona | Illumina MiSeq NextSeq 500, HiSeq 2500 | https://research.arizona.edu/facilities/core-facilities/ua-genetics-core-uagc |
|  | Lubbock, TX | Research and Testing Laboratory (RTL Genomics) | Illumina MiSeq, HiSeq, NovaSeq 6000; PacBio Sequel; Ion Torrent Proton, Ion Torrent PGM | <https://www.medicalbiofilm.org/next-generation-sequencing-service.html>  https://rtlgenomics.com/sequencing |
|  | Dallas, TX | Genomic Core Facility University of Texas Southwestern Medical Center | Illumina NextSeq 550, MiSeq Dx | https://www.utsouthwestern.edu/research/core-facilities/genomics/ |
|  | Dallas, TX | University of Texas Southwestern Medical Center | Illumina MiSeq, 3x NextSeq 500, NextSeq 2000; PacBio Sequel I | https://www.utsouthwestern.edu/research/core-facilities/next-generation-sequencing/ |
|  | Austin, TX | Center for Biomedical Research Support; The University of Texas at Austin | Illumina MiSeq, NextSeq 500, NovaSeq 6000 | https://research.utexas.edu/cbrs/cores/genomics/resources/methods-and-instruments/ |
|  | San Antonio, TX | The University of Texas at San Antonio | Illumina MiSeq | https://research.utsa.edu/cores/genomics/ |
|  | College Station, TX | Texas A&M Institute for Genome Sciences and Society (TIGSS) | Illumina MiSeq, NextSeq 500; Oxford Nanopore MinION | https://genomics.tamu.edu/genomics-core/ |
|  | College Station, TX | AgriLife Genomics and Bioinformatics Services | Illumina iSeq, MiSeq, NovaSeq 6000 | https://www.txgen.tamu.edu/faq/firststep/ |
|  | Denton, TX | The University of North Texas Genomics Center Core Facility | Illumina MiSeq, NextSeq 500 | https://research.unt.edu/research-units-facilities/university-managed-facilities/genomics-center/services |
|  | Arlington, TX | North Texas Genome Center; The University of Texas at Arlington | Illumina iSeq, MiSeq, NovaSeq 6000 | https://www.northtexasgenomecenter.com/sequencing/ |
|  | Houston, TX | Houston Methodist Hospital – Texas Medical Center | Illumina MiSeq; Ion Torrent PGM | https://www.houstonmethodist.org/molecular/ |
|  | Houston, TX | The Advanced Technology Genomics Core; MD Anderson Cancer Center | Illumina iSeq100, MiSeq, NextSeq 500, HiSeq 4000, 2x NovaSeq 6000 | https://www.mdanderson.org/research/research-resources/core-facilities/advanced-technology-genomics-core/services-and-fees/illumina-next-generation-sequencing.html#nextgen |
|  | Baton Rouge, LA | College of Science Genomics Core; Louisiana State University | Ion Torrent S5 | https://genomics.lsu.edu/genomics-services-ngs.php#iontorrents5 |
|  | Jupiter, FL | The Herbert Wertheim UF Scripps Institute for Biomedical Innovation & Technology | Illumina MiSeq, NextSeq 500 | https://scripps.ufl.edu/cores-and-technologies/genomics-core/ |
|  | Gainesville, FL | Interdisciplinary Center for Biotechnology Research; the University of Florida | Illumina iSeq, MiSeq, NovaSeq 6000; PacBio Sequel IIe; Oxford Nanopore PromethION, MinION | https://biotech.ufl.edu/next-gen-dna/ |
|  | Miami, FL | John P. Hussman Institute for Human Genomics (HIHG) | Illumina MiSeq, NextSeq, NovaSeq 6000 | https://med.miami.edu/centers-and-institutes/hihg/research-centers/center-for-genome-technology/sequencing-core |
|  | La Jolla, CA | The Sanford Burnham Prebys | Illumina NextSeq 500 | https://www.sbpdiscovery.org/biomedical-research/shared-resources/genomics-and-spatial-transcriptomics |
|  | Atlanta, GA | Otogenetics | Illumina MiSeq, HiSeq 2500 | https://www.otogenetics.com/hiseq2500-miseq-lane-rental/ |
|  | Winston-Salem, NC | School of Medicine; Wake Forest University | Illumina 2x MiSeq, 2x NextSeq 500, NextSeq 550, NovaSeq 6000 | https://school.wakehealth.edu/research/institutes-and-centers/comprehensive-cancer-center/center-for-cancer-genomics-and-precision-oncology |
|  | Chapel Hill, NC | School of Medicine; University of North Carolina | Illumina MiSeq, NextSeq 2000, NovaSeq 6000; Oxford Nanopore GridION; Bionano Saphyr | https://www.med.unc.edu/genomics/technology/ |
|  | Knoxville, TN | The University of Tennessee Knoxville | Illumina MiSeq, NovaSeq 6000; Oxford Nanopore MinION | https://ceb.utk.edu/high-throughput-sequencing/ |
|  | Nashville, TN | Vanderbilt University Medical Center | Illumina MiSeq, NextSeq 500, NovaSeq 6000 | https://www.vumc.org/vantage/next-gen-sequencing |
|  | Durham, NC | Duke University School of Medicine | MiSeq, NextSeq 1000, NovaSeq 6000; Oxford Nanopore GridION | https://biostat.duke.edu/research/center-genomic-and-computational-biology/gcb-core-facilities/sequencing-and-genomic-2 |
|  | Atlanta, GA | Emory University | Illumina MiSeq, NovaSeq 6000 | http://www.yerkes.emory.edu/nhp_genomics_core/services/sequencing.html |
|  | Atlanta, GA | The Emory Integrated Genomics Core | Illumina MiSeq, NextSeq 550 | https://www.cores.emory.edu/eigc/resources/methods.html |
|  | Athens, GA | The Georgia Genomics and Bioinformatics Core; the University of Georgia | Illumina MiSeq, NextSeq 500, NextSeq 2000; PacBio Sequel; Oxford Nanopore MinIon | https://dna.uga.edu/ |
|  | Huntsville, AL | HudsonAlpha Institute for Biotechnology | Illumina NovaSeq 6000; PacBio Sequel II, Sequel IIe, Revio | https://www.hudsonalpha.org/gsc/capabilities/ |
|  | Los Angeles, CA | Molecular Genomics Core; University of South Carolina | Illumina MiSeq, NextSeq 2000 | <https://uscnorris.com/Core/MolGen/>;  https://uscnorriscancer.usc.edu/wp-content/uploads/2023/03/MGC-Pricing_updated-4_1_2023.pdf |
|  | Richmond, VA | Virginia Commonwelath University | Illumina MiSeq, NovaSeq 2000 | https://research.vcu.edu/cores/genomics/services/ |
|  | Silver Spring, MD | Walter Reed Army Institute of Research | Illumina MiSeq, NextSeq; PacBio RSII | https://wrair.health.mil/Portals/87/MRSN_Specimen_Submission_Manual.pdf |
|  | Bethesda, MD | Uniformed Services University | Illumina NextSeq 500, 10x HiSeq X Ten, HiSeq 3000, 2x NovaSeq 6000 | https://cmph.usuhs.edu/bioinformatics-genomics |
|  | Kansas City, MO | CBRNE Tech Index | Ion Torrent S5 | https://www.cbrnetechindex.com/Biological-Detection/Technology-BD/Molecular-BD-T/Next-Generation-Sequencing-BD-M |
|  | Boston, MA | Harvard T.H. Chan School of Public Health; Center for Computational and Integrative Biology; Massachusetts General Hospital | Illumina MiSeq; Ion Torrent PGM; Oxford Nanopore | <https://www.hsph.harvard.edu/program-genetic-epidemiology-and-statistical-genetics/genotyping-core-facility/>  <https://dnacore.mgh.harvard.edu/new-cgi-bin/site/pages/next_generation_sequencing_main.jsp>  https://dnacore.mgh.harvard.edu/new-cgi-bin/site/pages/rapid_plasmid_sequencing_main.jsp |
|  | Boston, MA | Biopolymers Facility; Harvard Medical School | Illumina 3x MiSeq, 4x Illumina NextSeq 500, NovaSeq 6000 | https://genome.med.harvard.edu/services/nextgen/ViewOverview.action |
|  | Boston, MA | Mass General Hospital | Illumina MiSeq, NextSeq 2000 | http://nextgen.mgh.harvard.edu/Pricing.html |
|  | Boston, MA | Mass General Brigham | Illumina NextSeq 550, NovaSeq 6000 | https://www.massgeneralbrigham.org/en/research-and-innovation/centers-and-programs/personalized-medicine |
|  | Worcester, MA | UMass Chan Medical School | Illumina MiSeq, MiSeq Dx, NovaSeq 6000 | https://www.umassmed.edu/nemo/deep-sequencing-services/ |
|  | Bethesda, MD | National Institute of Diabetes and Digestive and Kidney Diseases (NIDDK) | Illumina MiSeq, NextSeq 550, HiSeq 3000 | https://www.niddk.nih.gov/research-funding/at-niddk/cores-support-services/genomics-core |
|  | Baltimore, MD | Center for Inherited Disease Research; John Hopkins University | Illumina NovaSeq 6000 | https://cidr.jhmi.edu/SequencingProducts.html |
|  | Rockville, MD | NIH Intramural Sequencing Center (NISC) | Illumina MiSeq, NextSeq 550, NovaSeq 6000; PacBio Sequel II; Oxford Nanopore GridION, PromethION | https://www.nisc.nih.gov/platforms.htm |
|  | Baltimore, MD | Genetic Resources Core Facility, Johns Hopkins University School of Medicine | Illumina MiSeq, NovaSeq 6000; Oxford Nanopore GridION | https://grcf.jhmi.edu/sequencing/ |
|  | Baltimore, MD | Johns Hopkins Medicine | Illumina MiSeq, NextSeq 500, HiSeq, NovaSeq; Oxford Nanopore MinION | https://www.hopkinsmedicine.org/institute_basic_biomedical_sciences/services/single-cell-sequencing-transcriptomics-core/ |
|  | Baltimore, MD | Institute for Genome Sciences; School of Medicine; University of Maryland | Illumina iSeq, MiSeq, NextSeq 550, NextSeq 550Dx, NovaSeq 6000; 3x PacBio Sequel II/IIe; Oxford Nanopore MinION | https://marylandgenomics.org/platforms/index.php |
|  | New York, NY | Memorial Sloan Kettering Cancer Center | Illumina MiSeq, NextSeq 500, NextSeq 2000, NovaSeq 6000; Oxford Nanopore MinION, PromethION | https://www.mskcc.org/research-advantage/core-facilities/integrated-genomics-operation-igo/igo-services |
|  | New York, NY | New York Genome Center | Illumina 12x HiSeq 2500, 10x HiSeq X | https://www.nygenome.org/wp-content/uploads/NYGC-nopricing.pdf |
|  | New York, NY | Center for Genomics and Systems Biology; New York University | Illumina MiSeq, NextSeq 500, NovaSeq 6000 | https://gencore.bio.nyu.edu/platforms-specifications/ |
|  | Storrs, CT | Institute for Systems Genomics; University of Connecticut | Illumina 2x MiSeq, NextSeq 550, NovaSeq 6000; Oxford Nanopore MinION, PromethION; Bionano Irys | https://cgi.uconn.edu/instrumentation/ |
|  | New York, NY | Rockefeller University Genomics Resource Center | Illumina MiSeq, NextSeq 500, NovaSeq 6000 | https://www.rockefeller.edu/genomics/instruments/ |
|  | New York, NY | Columbia Genome Center; Columbia University | Illumina MiSeq, 3x NextSeq 500/550, NovaSeq 6000 | https://systemsbiology.columbia.edu/genome-center/sequencing-and-analysis |
|  | Newark, NJ | New Jersey Medical School | Illumina MiniSeq, MiSeq, NextSeq 500, NovaSeq 6000 | <https://research.njms.rutgers.edu/genomics/doc/Instruments.pdf> |
|  | Providence, RI | Brown University | Illumina NextSeq 550 | https://biomedcorefacilities.brown.edu/genomics-facility/next-generation-sequencing |
|  | Kingston, RI | University of Rhode Island | Illumina MiSeq | https://web.uri.edu/riinbre/gsc-move/ |
|  | New Haven, CT | Yale Center for Genome Analysis | Illumina MiSeq, HiSeq 2500, HiSeq 4000, NovaSeq 6000; PacBio Sequel II | https://medicine.yale.edu/keck/ycga/sequencing/ |
|  | New Haven, CT | Genomics and Bioinformatics Core; Yale School of Medicine | Illumina HiSeq 4000 | https://medicine.yale.edu/stemcell/coreservices/corelabs/genomics/ |
|  | Boston, MA | Dana-Farber Cancer Institute | Illumina MiniSeq, MiSeq, NextSeq 500, NovaSeq 6000 | http://mbcf.dfci.harvard.edu/instrumentation |
|  | Boston, MA | Tufts University | Illumina MiSeq, NextSeq 550, HiSeq 2500, NovaSeq 6000 | http://tucf-genomics.tufts.edu/home/services |
|  | Cambridge, MA | Broad Institute Genomic Services | Illumina 9x MiSeq, 2x NextSeq 500, 9x HiSeq 2500, 17x HiSeq X10, 30x NovaSeq 6000, 5x NovaSeq X; PacBio Sequel I, Sequel II, Revio; Oxford Nanopore PromethION | <https://twitter.com/BroadGenomics>  https://cores.catalyst.harvard.edu/?core_id=218&uri_id=0000012e-6e00-af62-55da-381e80000000&category_id=7&navMode=cat |
|  | Cambridge, MA | Partners HealthCare Personalized Medicine | Illumina MiSeq, HiSeq 2500 | https://personalizedmedicine.partners.org/Translational-Genomics-Core/Services/default.aspx |
|  | Cambridge, MA | BioMicro Center; Massachusetts Institute of Technology | Illumina 2x MiSeq, 2x NextSeq 500, NovaSeq 6000; PacBio Sequel I; Oxford Nanopore PromethION | https://openwetware.org/wiki/BioMicroCenter:Sequencing |
|  | Cambridge, MA | Whitehead Institute | Illumina MiSeq, NovaSeq 6000 | https://wi.mit.edu/core-facilities/genomecore |
|  | Boston, MA | Boston Children’s Hospital | Illumina NextSeq 500; Bionano Saphyr | https://www.childrenshospital.org/research/cores/molecular-genetics-core/equipment |
|  | Boston, MA | Harvard Bauer Core | Illumina MiSeq, NextSeq 500, NextSeq 1000, NovaSeq 6000; PacBio Sequel IIe; Oxford Nanopore MinION, PromethION | <https://bauercore.fas.harvard.edu/instruments>  https://bauercore.fas.harvard.edu/instruments-0 |
|  | Philadelphia, PA | Perelman School of Medicine; University of Pennsylvania | Illumina MiSeq, NextSeq, NovaSeq 6000; Ion Torrent PGM, S5; Oxford Nanopore MinION | https://genetics.med.upenn.edu/cores/pgsc/ |
|  | Grand Rapids, MI | Van Andel Institute | Illumina NextSeq 500, NovaSeq 6000 | https://genomicscore.vai.org/?tabUrl=equipment |
|  | Burlington, VT | University of Vermont Cancer Cente | lumina MiniSeq, MiSeq, HiSeq 1500/2500; Oxford Nanopore MinION, GridION, MK1C | https://www.med.uvm.edu/vigr/dnaanalysis |
|  | Rensselaer, NY | Center for Functional Genomics; University at Albany | Illumina NextSeq 2000; Oxford Nanopore MinION | https://www.albany.edu/genomics/ngs.html |
|  | University Park, PA | The Huck Institutes of the Life Sciences, University Pennsylvania | Illumina MiSeq, NextSeq 2000, HiSeq 2500; PacBio Sequel IIe | https://www.huck.psu.edu/core-facilities/genomics-core-facility/instrumentation/instrumentation-list |
|  | Cleveland, OH | Lerner Research Institute; Cleveland Clinic | Illumina MiSeq, NovaSeq 6000 | https://www.lerner.ccf.org/cores/genomics/#core-equipment |
|  | Cleveland, OH | Case Western Reserve University | Illumina MiSeq, NextSeq 550 | https://case.edu/medicine/genomics/our-services/next-generation-sequencing |
|  | Albany, NY | Department of Health, Wadsworth Center | Illumina MiSeq; Ion Torrent PGM | https://www.wadsworth.org/research/cores/applied-genomics/next-gen-sequencing |
|  | Buffalo, NY | Jacobs School of Medicine and Biomedical Sciences; University at Buffalo | Illumina MiSeq, Illumina HiSeq 2500 | https://medicine.buffalo.edu/research/core_facilities.host.html/content/shared/smbs/core_resources/nextgen.detail.html |
|  | Ann Arbor, MI | Daicel Arbor Biosciences | Illumina MiSeq, NovaSeq; Ion Torrent Proton; BacBio Sequel II; Oxford Nanopore | https://arborbiosci.com/genomics/ngs-services/myreads/ |
|  | Kansas City, MO | Children’s Mercy Research Institute | Illumina 2x MiSeq, NovaSeq 2000, NovaSeq 6000; PacBio Sequel IIs | https://www.childrensmercy.org/childrens-mercy-research-institute/research-areas/genomic-medicine-center/genomic-medicine-center-facilities/ |
|  | Chicago, IL | University of Chicago | Illumina MiSeq, NextSeq 500, HiSeq 2500, HiSeq 4000, NovaSeq X | <https://microbiome.uchicago.edu/node/711>  https://voices.uchicago.edu/genomicsfacility/services/ |
|  | Cincinnati, OH | University of Cincinnati College of Medicine | Illumina NextSeq 2000 | https://med.uc.edu/docs/default-source/environmental-health-docs/genomics-core/2021-10-05_flier_ges-core.pdf?sfvrsn=3de43114_4 |
|  | Cincinnati, OH | Cincinnati Children's Hospital Medical Center | Illumina NovaSeq 6000 | https://www.cincinnatichildrens.org/research/cores/mgml |
|  | Iowa City, IA | Roy J. Carver Center for Genomics; University of Iowa | Illumina iSeq, NovaSeq 6000 | https://biology.uiowa.edu/ccg/ccg-services |
|  | Iowa City, IA | Iowa Institute of Human Genetics | Illumina MiSeq, NovaSeq 6000; Oxford Nanopore MinION | <https://medicine.uiowa.edu/humangenetics/genomics-division/genome-sequencing/sequencers>  https://medicine.uiowa.edu/humangenetics/genomics-division/genome-sequencing/long-read-sequencing |
|  | Omaha, NE | University of Nebraska Medical Center | Illumina MiSeq, NextSeq 550, NovaSeq 6000 | https://www.unmc.edu/vcr/cores/vcr-cores/genomics/next-generation/index.html |
|  | Columbus, OH | Nationwide Children’s Hospital | Illumina MiniSeq, MiSeq, HiSeq 4000, NovaSeq 6000 | https://www.nationwidechildrens.org/specialties/institute-for-genomic-medicine/genomic-services |
|  | Milwaukee, ‎WI | Medical College of Wiskonsin | Illumina MiSeq, NextSeq 500, HiSeq 2000, HiSeq 2500; PacBio Sequel II | <https://www.mcw.edu/-/media/MCW/Departments/Biochemistry/Program-in-Chemical-Biology/PCB-Equipment.pdf>  https://www.mcw.edu/-/media/MCW/Departments/Research-Resources/Core-Descriptions/Core-Description-Molecular-Biology-Core.pdf |
|  | Marshfield, WI | PreventionGenetics | Illumina NovaSeq 6000 | https://www.preventiongenetics.com/pgnome/ |
|  | Athens, OH | Ohio University Genomics Facility | Illumina MiSeq, Ion Torrent PGM | https://www.ohio.edu/cas/genomics/services-resources/next-generation-sequencing |
|  | Columbus, OH | Comprehensive Cancer Center; Ohio State University | Illumina iSeq, 2x MiniSeq, 3x MiSeq, 2x NovaSeq 6000; PacBio Sequel | https://cancer.osu.edu/for-cancer-researchers/resources-for-cancer-researchers/shared-resources/genomics/equipment |
|  | Columbus, OH | Molecular and Cellular Imaging Center; Ohio State University | Illumina 2x MiSeq; Oxford Nanopore MinION | https://mcic.osu.edu/genomics/illumina-sequencing |
|  | Lemont, IL | Argonne National Laboratory | Illumina MiSeq, HiSeq 2500 | https://www.anl.gov/bio/environmental-sample-preparation-and-sequencing-facility |
|  | St. Louis, MO | Genome Technology Access Center; Washington University in St. Louis | Illumina 5x NovaSeq 6000 | https://research.wustl.edu/core-facilities/genome-technology-access-center/ |
|  | St. Louis, MO | Genome Access Technology Center at the McDonnell Genome Institute (GTAC@MGI) | Illumina MiSeq, NovaSeq 6000, NovaSeq X; PacBio Sequel II, Revio; Oxford Nanopore PromethION; Bionano Saphyr | <https://gtac.wustl.edu/laboratory-services/long-read-seq-mapping/>  https://gtac.wustl.edu/laboratory-services/illumina-sequencing/ |
|  | East Lansing, MI | Michigan State University | Illumina MiSeq, NovaSeq 6000; Oxford Nanopore GridION | https://rtsf.natsci.msu.edu/genomics/pricing.aspx |
|  | Notre Dame, IN | University of Notre Dame | Illumina MiSeq, NextSeq 500 | https://genomics.nd.edu/services/illumina-sequencing/ |
|  | Chicago, IL | University of Illinois Chicago | Illumina MiSeq, MiniSeq, NextSeq 500; Oxford Nanopore MinION | https://rrc.uic.edu/cores/genome-research/instruments-sqc/ |
|  | Columbia, MO | University of Missouri | Illumina MiSeq, NovaSeq 6000 | https://dnacore.missouri.edu/ngs.html |
|  | West Lafayette, IN | Purdue University | Illumina MiSeq | https://www.purdue.edu/discoverypark/bioscience/facilities/core/genomics/instruments.php |
|  | Kansas City, MO | Stowers Institute | Illumina MiSeq, NextSeq 500, NextSeq 2000; Oxford Nanopore MinION | https://www.stowers.org/technology-centers/sequencing-and-discovery-genomics |
|  | Bloomington, IN | [INDIANA UNIVERSITY BLOOMINGTON](https://www.indiana.edu/)Indiana University Bloomington | Illumina MiSeq, NextSeq; Oxford Nanopore | https://cgb.indiana.edu/sequencing/index.html |
|  | Lincoln, NE | University of Nebraska – Lincoln | Oxford Nanopore GridION | https://biotech.unl.edu/single-cell-genomics#tab4 |
|  | Chicago, IL | Feinberg School of Medicine; Northwestern University | Illumina MiSeq, NextSeq 500, HiSeq 4000, NovaSeq 6000; Oxford Nanopore MinION, PromethION | https://www.cgm.northwestern.edu/cores/nuseq/services/next-generation-sequencing/index.html |
|  | Corvallis, OR | Oregon State University | Illumina MiSeq, NextSeq 2000; PacBio Sequel II | https://cqls.oregonstate.edu/core/sequencing |
|  | Eugene , OR | Genomics & Cell Characterization Core Facility; University of Oregon | Illumina MiSeq, NextSeq 500, NovaSeq 6000; PacBio Sequel II | <https://gc3f.uoregon.edu/illumina-sequencing#novaseq>  https://gc3f.uoregon.edu/pacbio-sequencing-2 |
| Canada | Calgary | Centre for Health Genomics and Informatics; University of Calgary | Illumina iSeq, MiSeq, NextSeq 500, NovaSeq 6000; Oxford Nanopore MinION | https://cumming.ucalgary.ca/research/cat/health-genomics/instrumentation |
|  | Edmonton | University of Alberta | Illumina MiSeq | https://www.ualberta.ca/medicine/research/corefacilities/hcacore/instruments.html |
|  | Hamilton | McMaster University | Illumina MiSeq, HiSeq 1500; Ion Torrent PGM, S5; PacBio Sequel I | <https://fhs.mcmaster.ca/mcmaster-genome-facility/>  https://synapseconsortium.com/directory/mcmaster-genomics-facility/ |
|  | London | London Regional Genomics Centre | Illumina MiSeq, NextSeq; Oxford Nanopore MinION | https://www.robarts.ca/lrgc/ |
|  | Montreal | Montreal Clinical Research Institute (IRCM) | Illumina MiSeq, NextSeq 500, NovaSeq 6000 | https://www.ircm.qc.ca/en/molecular-biology-and-functional-genomics |
|  | Montreal | The Institute for Research in Immunology and Cancer (IRIC) | Illumina MiSeq, NextSeq 500 | https://www.iric.ca/en/research/platforms-and-infrastructures/genomics |
|  | Ottawa | Ottawa Hospital Research Institute | Illumina MiniSeq, NextSeq 500 | https://www.ohri.ca/stemcore/LaboratoryServices/Illumina.aspx |
|  | Toronto | The Centre for Applied Genomics; Hospital for Sick Children (SickKids) | Illumina MiSeq, NovaSeq 6000; PacBio Sequel IIe; Oxford Nanopore PromethION | http://www.tcag.ca/facilities/dnaSequencingSynthesis.html#5 |
|  | Toronto | Ontario Institute for Cancer Research | Illumina MiSeq, NextSeq500, NovaSeq 6000; Oxford Nanopore MinION, PromethION | https://genomics.oicr.on.ca/services/ |
|  | Toronto | Donnelly Sequencing Centre; University of Toronto | Illumina MiSeq, NextSeq, NovaSeq 6000 | https://thedonnellycentre.utoronto.ca/what-we-offer |
|  | Vancouver | BC Cancer Research Centre | Illumina MiSeq, NextSeq 500, HiSeq X, NovaSeq 6000; Oxford Nanopore MinION, PromethION; MGI DNBSEQ-G400 | https://bcgsc.ca/about-us/technology-platforms |
